# Supplementary material for: Systematic Transcriptome and Regulatory Network Analyses Reveal the Hypoglycemic Mechanism of Dendrobium fimbriatum
Source: Mol Ther Nucleic Acids. 2019 Nov 11;19:1–14. doi: 10.1016/j.omtn.2019.10.033 (PMC6909217; doi:10.1016/j.omtn.2019.10.033)
Supplement: Document S1. Figures S1–S7 and Tables S1 and S2 [file mmc1.pdf]

**OMTN, Volume *19***

## Supplemental Information

# Systematic Transcriptome and Regulatory Network Analyses Reveal the Hypoglycemic Mechanism of *Dendrobium fimbriatum*

**Qiong Zhang, Jing Li, Mei Luo, Gui-Yan Xie, Weiwei Zeng, Yuxin Wu, Yanhong Zhu, Xiangliang Yang, and An-Yuan Guo**

## Supplementary Figures and Tables

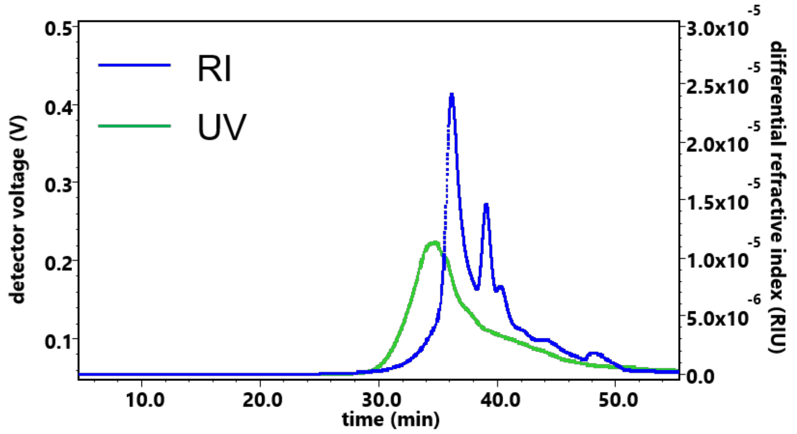


Supplementary Figure 1 | High performance size exclusion chromatography (HPSEC)/refractive index (RI) of *D. fimbriatum*.

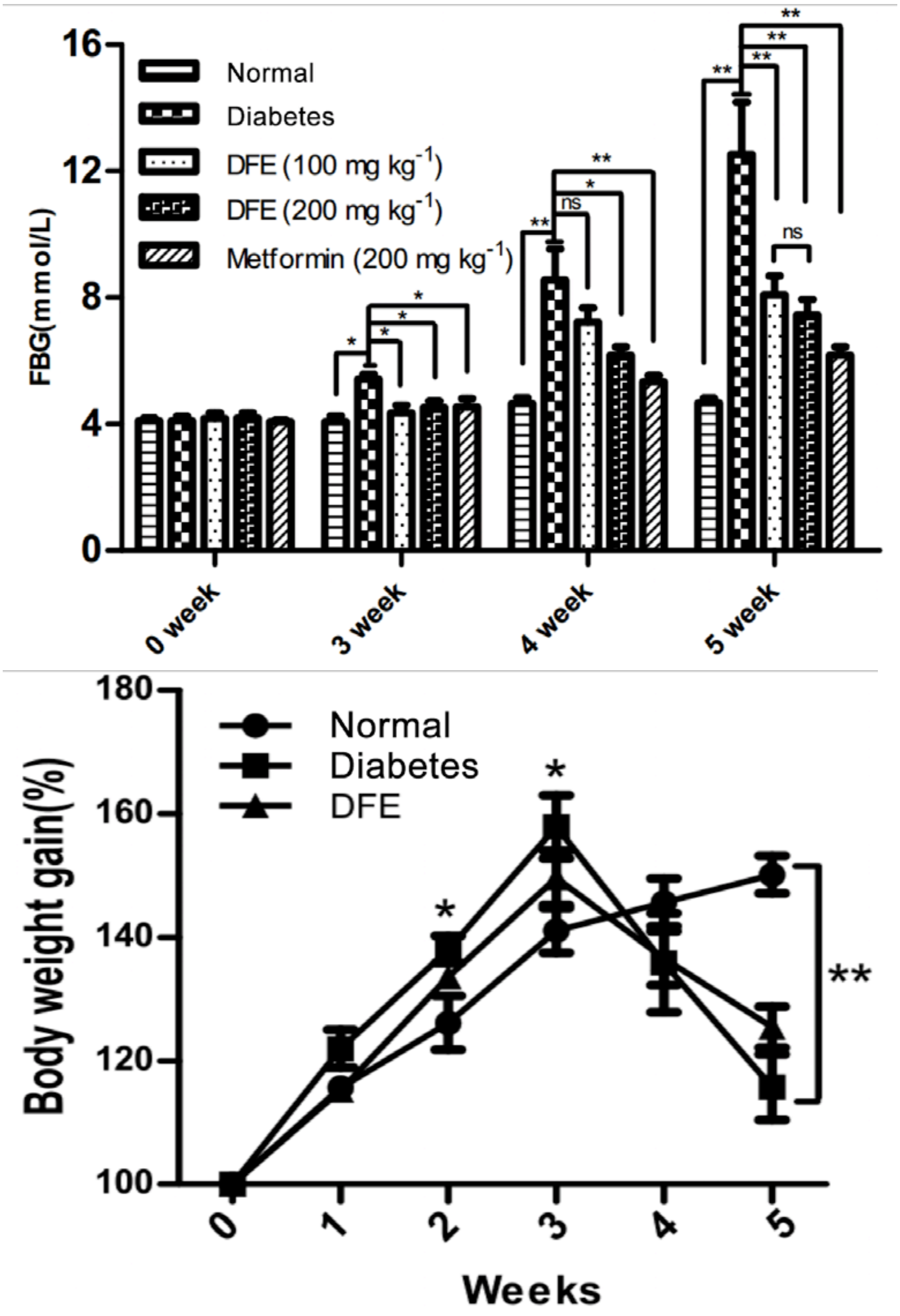


Supplementary Figure 2 | The dose effect of DFE alleviating diabetes symptom and body weight gain on diabetes rats. All quantitative data are reported as means ± SEM. *P-value < 0.05 and **P-value < 0.01 were determined by one-way ANOVA followed by Newman–Keuls post hoc tests. The ns represents no-significance.

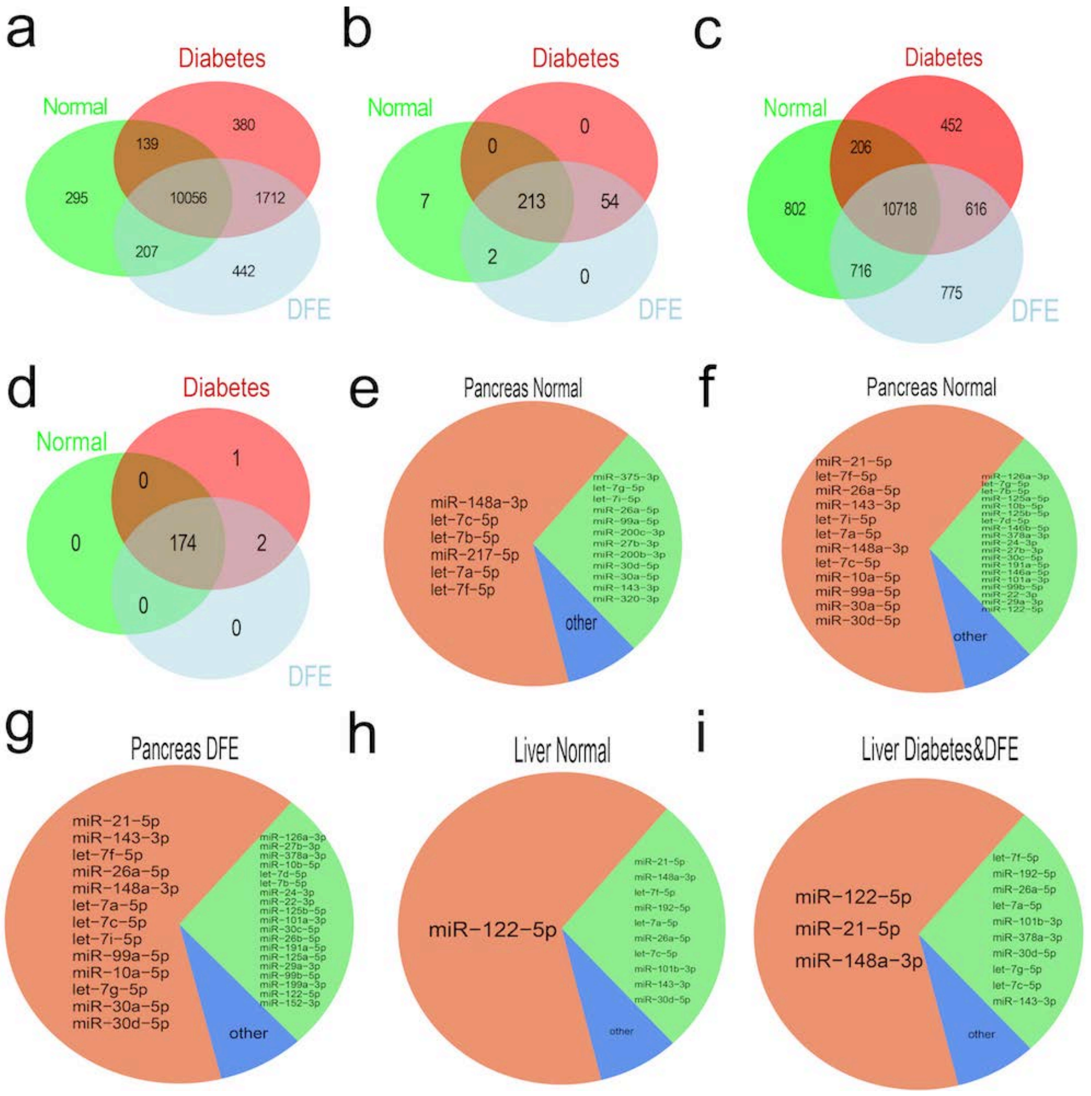


Supplementary Figure 3 | Transcriptome profiles of RNA-Seq and miRNA-Seq in the pancreas and liver across the normal, diabetes and diabetes-DFE groups. (A) Genes with FPKM>1 in the pancreas among the three groups; (B) miRNAs with TPM>10 in the pancreas among the three groups; (C) Genes with FPKM>1 in the liver among the three groups; (D) MiRNAs with TPM>10 in the liver among the three groups; (E-G) Component percentage of expressed miRNAs in the pancreas of the three groups, yellow: 70%, green: 20%, blue: 10%; (H, I) Component percentage of expressed miRNAs in the liver of the three groups.

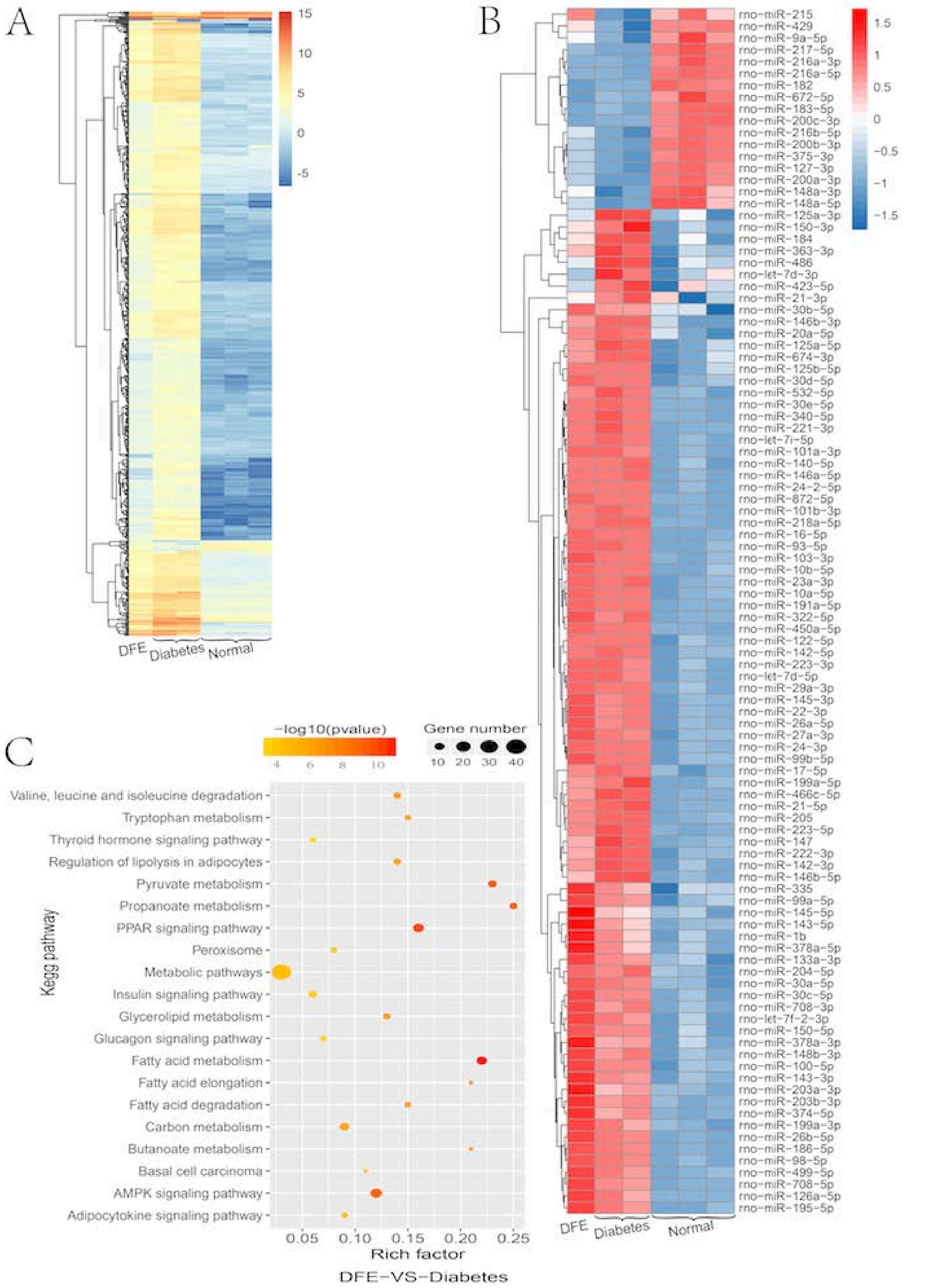


Supplementary Figure 4 | Heatmap of differentially expressed miRNAs in the pancreas and KEGG enrichment result of up-regulated DEGs in the comparison of diabetes-DFE-VS-diabetes. (A) Heatmap for 588 DEGs with opposite expression profiles in the comparisons of diabetes-DFE-VS-diabetes and diabetes -VS-normal; (B) Heatmap of DEMs in the pancreas among the three groups; (C) Top 20 terms of KEGG enrichment results in the comparison of diabetes-DFE-VS-diabetes.

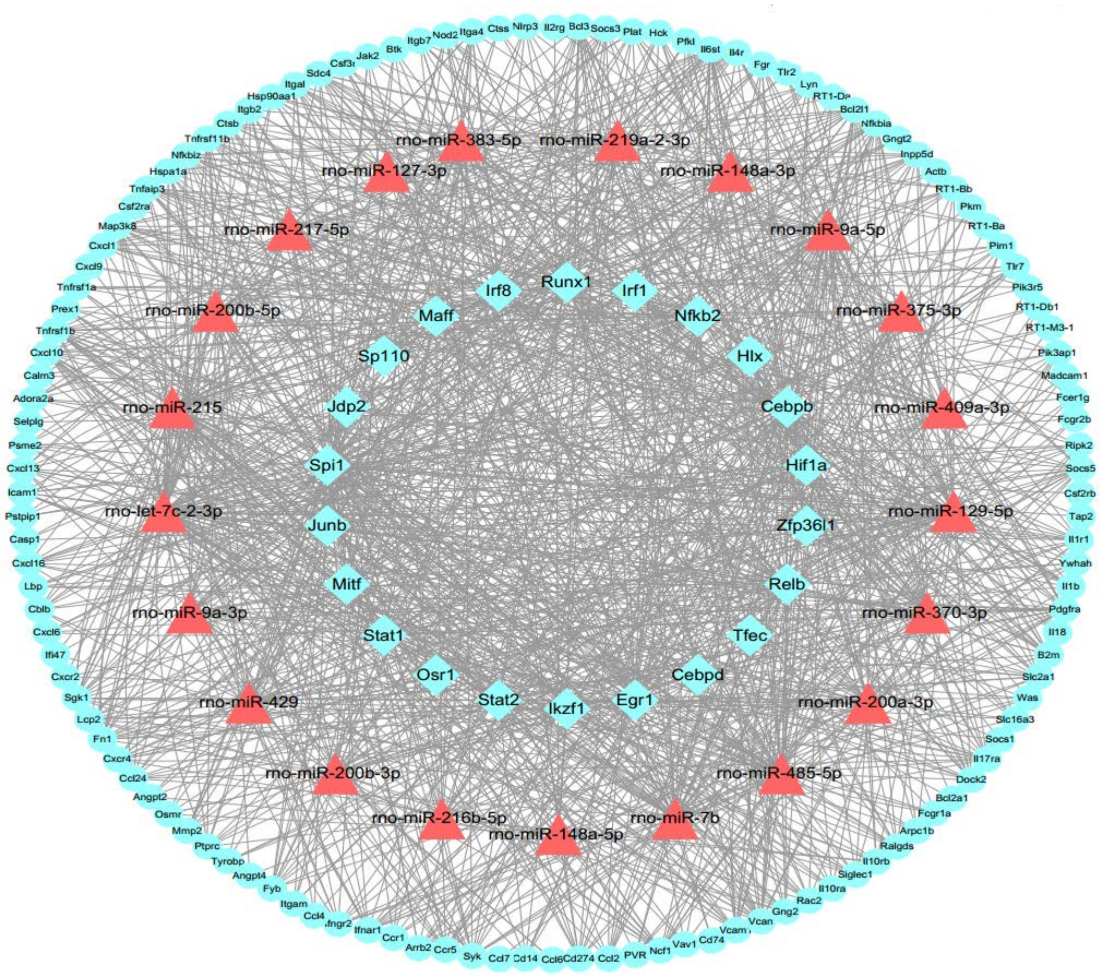


Supplementary Figure 5 | miRNAs-TFs-genes regulatory network involved in the effects of DFE on alleviating inflammation and apoptosis in the diabetic pancreas. Blue rectangles: TFs; red triangles: miRNAs; blue cycles: NON-TF genes.

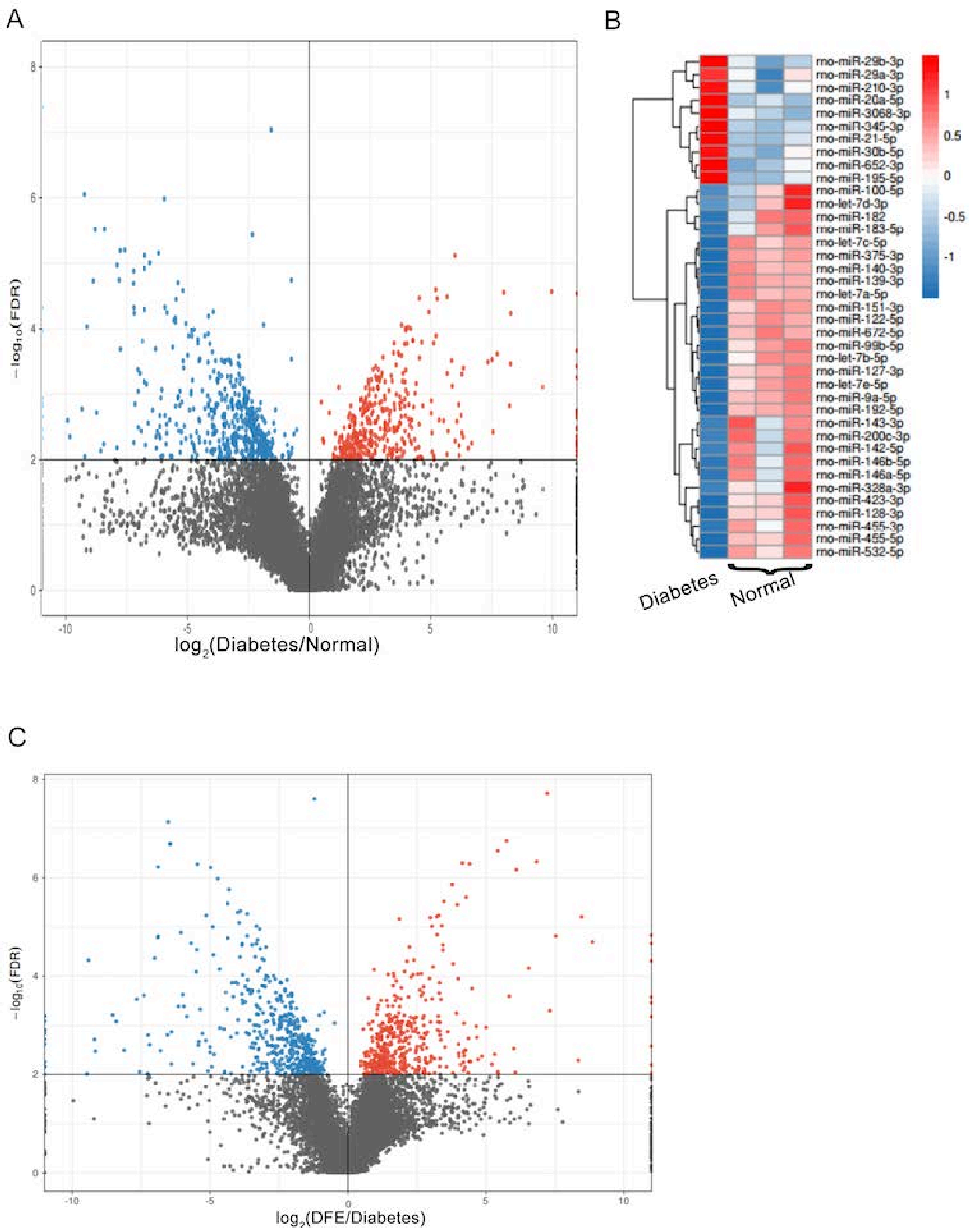


Supplementary Figure 6 | Expression profiling of DEGs and DEMs of the comparisons of diabetes-VS-normal and diabetes-DFE-VS-diabetes in the liver. (A) Volcano plot for the genes in the comparison of diabetes-VS-normal groups; (B) Heatmap of the DEMs in the comparison of diabetes-VS-normal; (C) Volcano plot for the genes in the comparison of diabetes-DFE-VS-diabetes groups.

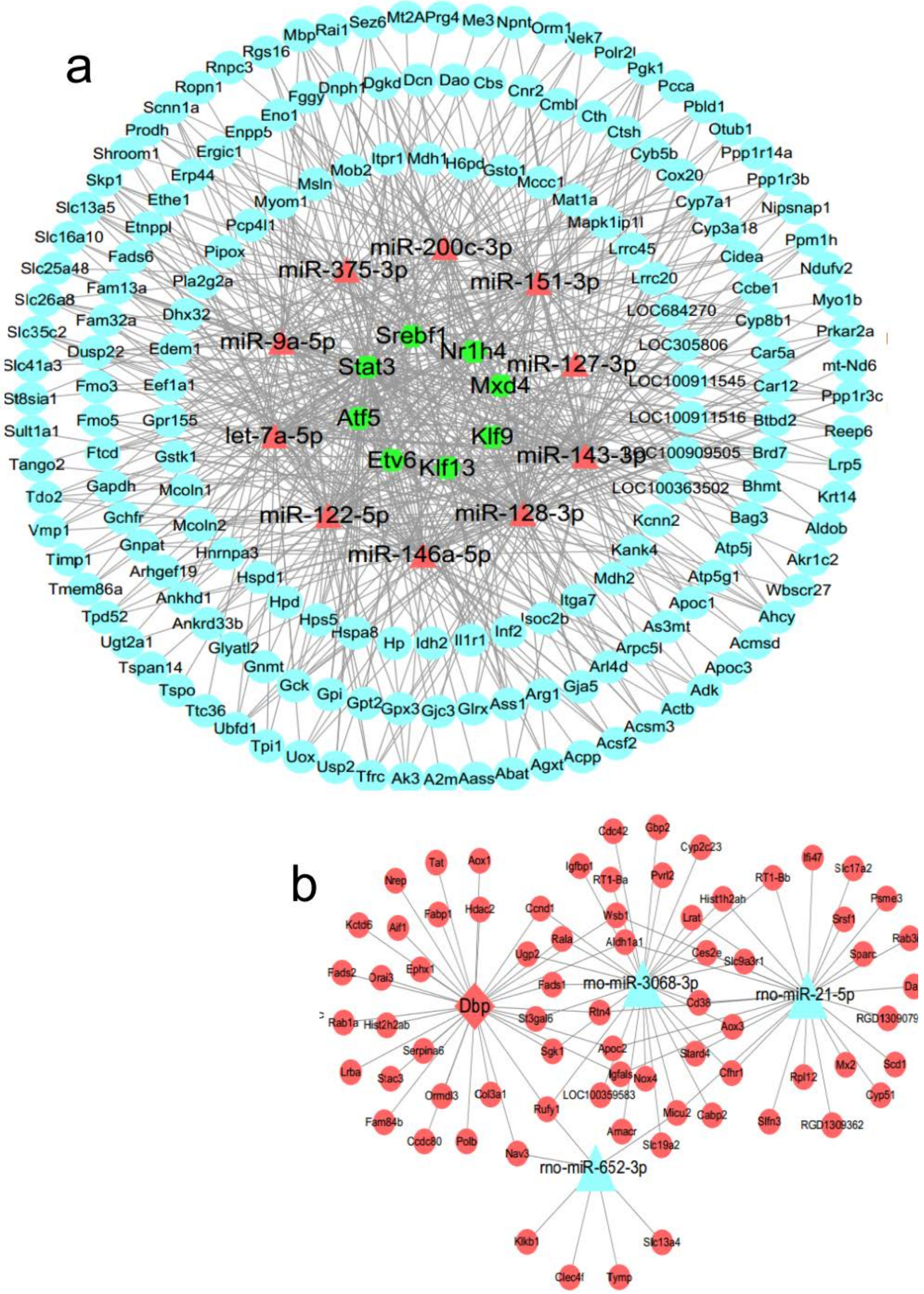


Supplementary Figure 7 | miRNA-TF regulatory network involved in the effects of DFE on alleviating lipid accumulation and hepatic apoptosis in the diabetic liver. Rectangles: TFs; triangles: miRNAs; cycles: NON-TF genes; red color: up-regulated; blue and green: down-regulated.

Supplementary Table 1. Basic statistics of RNA-Seq data for samples.

| **Sample Name** | **Tissues/ Treatment** | **Clean bases (G)** | **Q20 (%)** | **GC (%)** | **Align (%)** |
|---|---|---|---|---|---|
| A7-1-A | Liver/normal_3 | 11.32 | 97.13 | 49.64 | 93.64 |
| A9-1-A | Liver/normal_1 | 11.23 | 97.06 | 49.30 | 93.18 |
| A10-1-A | Liver/normal_2 | 11.12 | 97.77 | 48.99 | 94.46 |
| B11A | Liver/diabetes_1 | 10.81 | 96.42 | 50.03 | 94.14 |
| G41A | Liver/diabetes-DFE_1 | 11.00 | 97.66 | 49.59 | 95.15 |
| G71A | Liver/diabetes-DFE_2 | 10.82 | 97.59 | 49.58 | 95.34 |
| A1-NA | Pancreas/normal_1 | 10.21 | 96.79 | 54.63 | 96.43 |
| A2-LA | Pancreas/normal_2 | 10.04 | 96.48 | 53.80 | 96.06 |
| A6-NA | Pancreas/normal_3 | 10.10 | 96.48 | 54.61 | 96.51 |
| B32A | Pancreas/diabetes_1 | 10.81 | 97.22 | 49.47 | 94.52 |
| B62A | Pancreas/diabetes_2 | 10.80 | 96.87 | 48.63 | 94.30 |
| G42A | Pancreas/diabetes-DFE_1 | 11.05 | 97.25 | 49.34 | 94.94 |

Supplementary Table 2. Basic statistics of miRNA-Seq data for samples.

| **Sample Name** | **Tissues/ Treatment** | **Clean Reads (M)** | **Clean bases (G)** | **Q20 (%)** | **GC (%)** | **Align (%)** |
|---|---|---|---|---|---|---|
| A10-1-A | Liver/normal_3 | 10.59 | 0.24 | 99.59 | 43.93 | 94.92 |
| A7-1-A | Liver/normal_1 | 10.50 | 0.23 | 99.77 | 44.32 | 95.34 |
| A9-1-A | Liver/normal_2 | 10.53 | 0.23 | 99.50 | 44.03 | 95.74 |
| B11A | Liver/diabetes_1 | 10.41 | 0.23 | 99.52 | 46.36 | 94.21 |
| G41A | Liver/diabetes-DFE_1 | 10.40 | 0.24 | 99.66 | 46.25 | 94.32 |
| G71A | Liver/diabetes-DFE_2 | 10.36 | 0.23 | 99.55 | 44.80 | 94.08 |
| A1-NA | Pancreas/normal_1 | 10.54 | 0.25 | 99.74 | 53.99 | 96.83 |
| A2-LA | Pancreas/normal_2 | 8.14 | 0.18 | 99.85 | 53.32 | 96.92 |
| A6-NA | Pancreas/normal_3 | 10.37 | 0.24 | 99.83 | 54.78 | 97.34 |
| B32A | Pancreas/diabetes_1 | 11.98 | 0.27 | 99.56 | 44.53 | 93.78 |
| B62A | Pancreas/diabetes_2 | 10.48 | 0.23 | 99.58 | 43.66 | 96.82 |
| G42A | Pancreas/diabetes-DFE_1 | 11.95 | 0.26 | 99.65 | 43.77 | 96.46 |
