# Supplementary material for: Systematic Transcriptome and Regulatory Network Analyses Reveal the Hypoglycemic Mechanism of Dendrobium fimbriatum
Source: Mol Ther Nucleic Acids. 2019 Nov 11;19:1–14. doi: 10.1016/j.omtn.2019.10.033 (PMC6909217; doi:10.1016/j.omtn.2019.10.033)
Supplement: Document S2. Article plus Supplemental Information [file mmc2.pdf]

Molecular Therapy
Nucleic Acids
Original Article

Systematic Transcriptome and Regulatory Network Analyses Reveal the Hypoglycemic Mechanism of *Dendrobium fimbriatum*

Qiong Zhang,[1,3] Jing Li,[2,3] Mei Luo,[1] Gui-Yan Xie,[1] Weiwei Zeng,[2] Yuxin Wu,[2] Yanhong Zhu,[2] Xiangliang Yang,[2] and An-Yuan Guo[1]

[1]Department of Bioinformatics and Systems Biology, Key Laboratory of Molecular Biophysics of the Ministry of Education, College of Life Science and Technology, Huazhong University of Science and Technology, Wuhan, China; [2]National Engineering Research Center for Nano Medicine, College of Life Science and Technology, Huazhong University of Science and Technology, Wuhan, China

Type 2 diabetes (T2D) is a long-term metabolic disorder disease characterized by high blood sugar and relative lack of insulin. Previous studies have demonstrated that *Dendrobium* has potent glucose-lowing effects and may serve as add-ons or alternatives to classic medications for T2D prevention and treatment, but the underlying molecular mechanisms were still unclear. We performed biochemical and transcriptional profiling (RNA sequencing [RNA-seq] and microRNA sequencing [miRNA-seq]) analyses on the pancreas and liver of *Dendrobium fimbriatum* extract (DFE)-fed diabetic rats and control animals. Our sequencing and experimental data indicated that DFE significantly alleviated diabetes symptoms through inhibiting inflammation and preventing islet cell apoptosis in diabetic pancreas. Transcription factors in Stat/nuclear factor κB (NF-κB)/Irf families combined with miR-148a/375/9a served as key regulators in the inflammation and apoptosis pathways under DFE administration. Meanwhile, DFE improved the energy metabolism, lipid transport, and oxidoreductase activity in the liver, and thus decreased lipid accumulation and lipotoxicity-induced hepatocyte apoptosis. Our findings revealed that DFE may serve as a potential therapeutic agent to prevent T2D, and also showed the combination of transcriptome profiling and regulatory network analysis could act as an effective approach for investigating potential molecular mechanisms of traditional Chinese medicine on diseases.

INTRODUCTION

Diabetes mellitus (DM) is a worldwide metabolic disorder disease caused by pancreatic β cell dysfunction and insulin dysbiosis,[1] and mainly characterized by hyperglycemia and dyslipidemia.[2] Most hypoglycemic agents used for diabetes treatment, including synthetic insulin and western medicine, may bring about side effects, such as hypoglycemia and myocardial infarction.[3] Traditional Chinese medicines (TCMs) as health food resources could serve as an effective supplement for diabetes therapy.[4]

TCM has more than 2,000 years of history and has been widely used in clinical studies for diabetes therapy. *Dendrobium*, as a traditional edible and medicinal plant in the Chinese Pharmacopoeia (National Pharmacopoeia Committee, 2015), is widely distributed in the tropical and subtropical regions of Europe/Asia/Oceania. Previous studies have demonstrated that *Dendrobium* has potent glucose-lowering effects and may serve as add-ons or alternatives for the prevention and treatment of diabetes.[5–7] *Dendrobium* contains multiple active components, such as polysaccharides, alkaloids, and glycosides, among others, in which, the *Dendrobium* polysaccharides are the main components.[8] Pharmacology research has proved that *Dendrobium* and the polysaccharide extracts possessed hypoglycemic, hepatoprotective, and hypolipidemic effects.[9] *Dendrobium* could effectively reduce the levels of blood glucose, triglyceride, and serum glycosylated protein in hyperglycemic mouse and diabetic rat models.[10] Previous studies demonstrated that the *Dendrobium* polysaccharides could inhibit *JNK* phosphorylation and promote *AKT* ser^{473} phosphorylation in the islets tissue of diabetic rats.[11] However, rare research had systematically investigated the potential molecular mechanisms underlying the anti-hyperglycemia effects of *D. fimbriatum* on diabetes.

Next generation sequencing (NGS)-based transcriptome profiling could offer more comprehensive views for potential mechanisms

Received 22 June 2019; accepted 25 October 2019;
https://doi.org/10.1016/j.omtn.2019.10.033.

[3]These authors contributed equally to this work.

Correspondence: An-Yuan Guo, Department of Bioinformatics and Systems Biology, Key Laboratory of Molecular Biophysics of the Ministry of Education, College of Life Science and Technology, Huazhong University of Science and Technology, 1037 Luoyu Road, Wuhan 430074, China.
E-mail: guoay@hust.edu.cn
Correspondence: Yanhong Zhu, National Engineering Research Center for Nano Medicine, College of Life Science and Technology, Huazhong University of Science and Technology, 1037 Luoyu Road, Wuhan 430074, China.
E-mail: yhzhu@hust.edu.cn
Correspondence: Xiangliang Yang, National Engineering Research Center for Nano Medicine, College of Life Science and Technology, Huazhong University of Science and Technology, 1037 Luoyu Road, Wuhan 430074, China.
E-mail: yangxl@hust.edu.cn

This is an open access article under the CC BY license (http://creativecommons.org/licenses/by/4.0/).

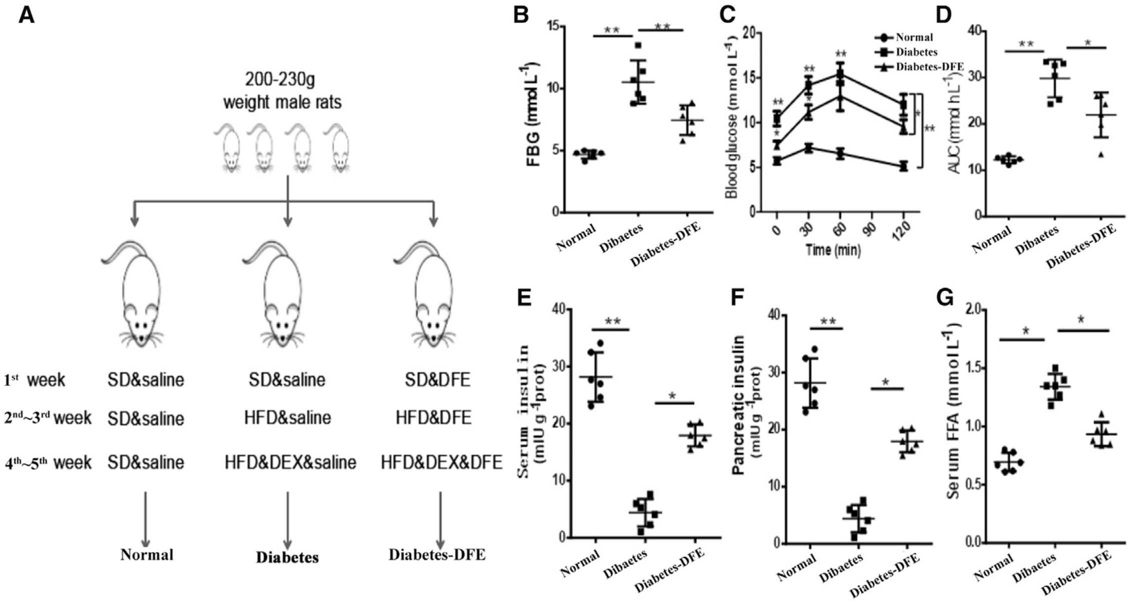

Figure 1. Modeling Process of the Experiment and Characteristic Signs of DM in Normal, Diabetic, and DFE Administration Rats (Diabetes-DFE)

(A) The design of this study. (B) Fasting blood glucose (FBG) levels of rats after experiments. (C) Blood glucose levels of different time points after experiments. (D) The value of AUCs of blood glucose level in (C) for each rat. (E) The levels of serum insulin in rats after experiments. (F) The levels of pancreatic insulin in rats after experiments. (G) The serum FFAs levels of rats after experiments. All quantitative data are means ± SEM. $^*p < 0.05$ and $^{**}p < 0.01$ were determined by one-way ANOVA followed by Newman-Keuls post hoc tests. DEX, dexamethasone; HFD, high-fat diet; SD, standard diet.

involved in diabetes and its complications in diabetic models or human samples.[12,13] Furthermore, transcription factor (TF) and microRNA (miRNA) as two major regulators of gene expression at transcriptional and post-transcriptional levels may form a feed-forward loop contributing to the development of diabetes.[14] However, few studies were conducted to explore the co-regulation of TFs and miRNAs on diabetic models, and rare studies focused on the molecular mechanisms of how *Dendrobium* showing hypoglycemic effects on diabetes.

In this study, we proved that the extracts of *D. fimbriatum* (DFEs) could increase the level of insulin and alleviate hyperglycemia in diabetic rats. To investigate potential molecular mechanisms of how the DFE regulates blood glucose, we performed transcriptome profiling (RNA sequencing [RNA-seq] and microRNA sequencing [miRNA-seq]) analysis and experimental validation on the pancreas and liver from DFE administration, diabetes, and normal rats. Our data imply that the DFE prevents β cell apoptosis and decreases hepatic lipid accumulation, which may be useful for the prevention and treatment of diabetes and its complications.

RESULTS

DFE Significantly Alleviates Hyperglycemia and Improves Glucose Tolerance in Diabetic Rats

A detailed experimental design was shown in the Figure 1A. Compared with the normal rats, high-fat diet (HFD) and dexamethasone (DEX) administration severely impaired the glucose tolerance capacity and elevated fasting blood glucose (FBG) in the diabetic rats (diabetes group; Figures 1B and 1D). The peak concentrations of blood glucose appeared at 60 mins after oral glucose intake and thereafter returned to basal values (Figure 1C). The area under the curve (AUC) of the blood glucose level (source data of Figure 1C, sampled from the time points of 0–120 mins) in the diabetic rats was significantly larger than others ($p < 0.01$; Figure 1D). Moreover, a decrease of insulin concentration and an increase of serum free fatty acids (FFAs) level were observed in the diabetic rats (Figures 1E–1G). These results indicated that the diabetic model was successfully constructed.

To evaluate the hypoglycemic effects of DFE on diabetes, two different doses of DFE (100 and 200 mg/kg) were orally administrated, and the metformin (200 mg/kg) was used for positive control (Figure S2). Oral administration of 100 and 200 mg/kg DFE showed a similar hypoglycemic effect on the diabetic rats (Figure S2), hence the dose of 100 mg/kg was selected for the further study, and the rats with oral DFE administration were classified to the diabetes-DFE group in this study. DFE administration significantly decreased FBG and the blood glucose level compared with the diabetic rats (Figures 1B and 1C), which implied that the DFE could improve the glucose tolerance. The AUC values of blood glucose level were markedly smaller with DFE administration compared with the diabetes group (Figure 1D). Meanwhile, the concentrations of serum and pancreatic insulin were both increased with DFE administration (Figures 1E and 1F), whereas the level of serum FFAs was decreased (Figure 1G). Combined with the results above, our data implied that DFE administration

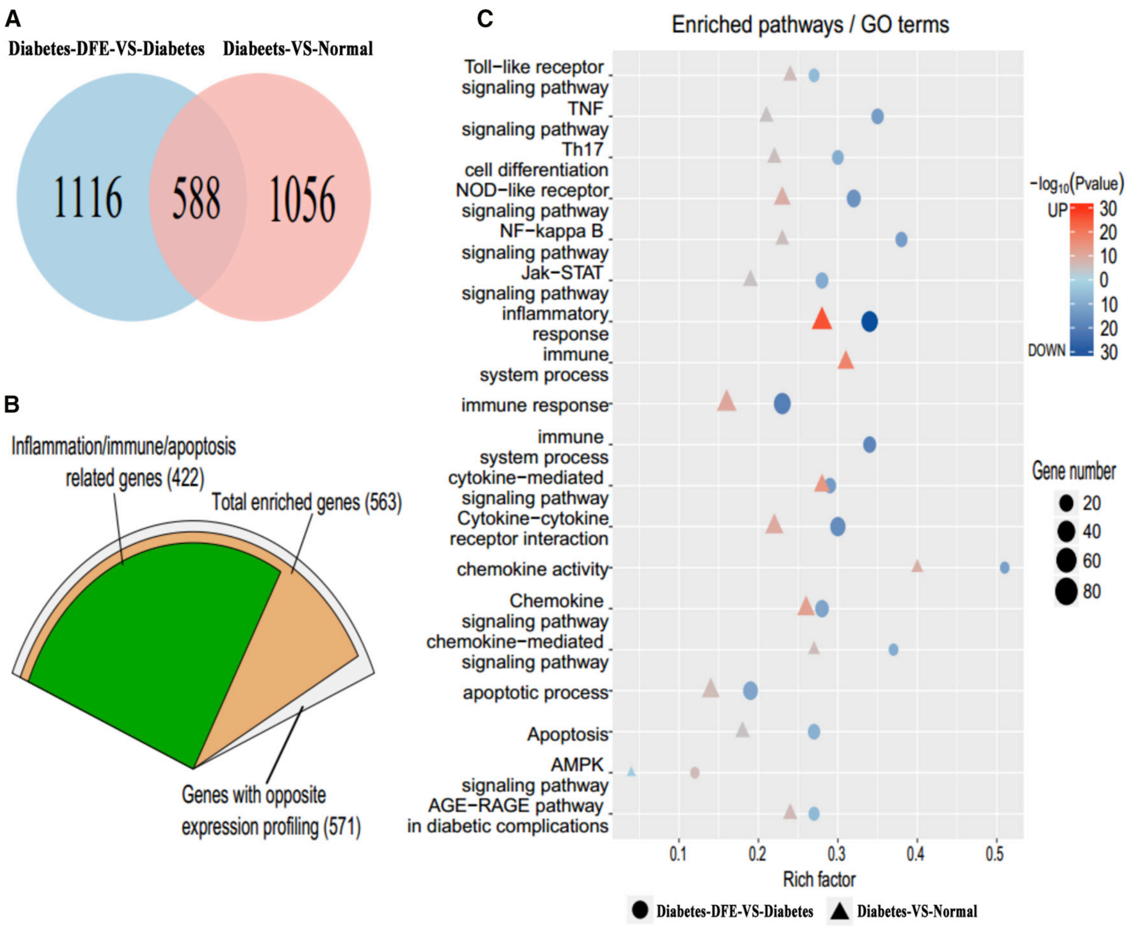

Figure 2. Transcriptome Profiling Analysis for the Pancreas across the Normal, Diabetes, and Diabetes-DFE Groups

(A) Common DEGs between the diabetes-VS-normal and diabetes-DFE-VS-diabetes comparisons. (B) Categories of the common DEGs between the diabetes-VS-normal and diabetes-DFE-VS-diabetes comparisons. (C) Functional enrichment results of DEGs in diabetes-VS-normal and diabetes-DFE-VS-diabetes comparisons.

could significantly increase the insulin concentration and lower the blood glucose level in the diabetic rats, which may thereby alleviate hyperglycemia.

Transcriptome Profiling of the Pancreas and Liver for Normal, Diabetes, and Diabetes-DFE Groups

To investigate potential molecular mechanisms underlying DFE alleviating hyperglycemia, we performed transcriptome sequencing (RNA-seq and miRNA-seq) for the pancreas and liver tissues in the three groups (normal, diabetes, and diabetes-DFE). The group description and basic statistics of NGS data were summarized in Tables S1 and S2. In total, we found 13,231 genes and 276 miRNAs expressed in the pancreas (Figures S3A and S3B), while 14,285 genes and 177 miRNAs were detected in the liver samples (Figures S3C and S3D). Meanwhile, several miRNAs, such as the *let-7* family members and *miR-148/miR-143*, were highly expressed in the pancreas and accounted for 70% of the total expression of all miRNAs (Figures S3E–S3G). A set of 12 miRNAs, including *miR-122*, *let-7a/b/c/f*, and *miR-148*, among others, accounted for 90% of total miRNA expressions in the liver samples (Figures S3H and S3I).

Bioinformatics Analysis Demonstrated that DFE Influenced Inflammatory-Related Processes in the Diabetic Pancreas

We detected 1,645 and 1,704 differentially expressed genes (DEGs) in the pancreas for diabetes-VS-normal and diabetes-DFE-VS-diabetes group comparisons, respectively. Among them, 588 DEGs showed the opposite expression pattern in the two comparisons, which indicated that these DEGs may attribute to the glucose-lowering effects of DFE in the pancreas (Figure 2A; Figure S4A). Interestingly, most of the 588 DEGs (97%, 571) were significantly downregulated in the diabetes-DFE-VS-diabetes comparison and upregulated in the diabetes-VS-normal comparison. Moreover, 74% of the 571 DEGs were strongly associated to inflammatory and immune processes (Figure 2B), which implied the main effects of DFE on the diabetic pancreas may focus on the anti-inflammation. Furthermore, we identified 70 and 59 differentially expressed miRNAs (DEMs) in the diabetes-VS-normal and

diabetes-DFE-VS-diabetes comparisons, respectively (Figures S4B and S4C).

Notably, 87% of DEGs significantly upregulated in the diabetes-VS-normal group comparison were enriched in inflammation and immune-related processes, including inflammatory and immune response, the cytokine and chemokine pathway, JAK-STAT/nuclear factor κB (NF-κB)/NOD-like/Toll-like receptor signaling pathways, and apoptosis pathways (Figure 2C). Interestingly, the significantly downregulated DEGs in the diabetes-DFE-VS-diabetes comparison were mainly involved in three biological categories, which were upregulated in the diabetes-VS-normal comparison: (1) cytokine receptor activity, (2) inflammatory and immune processes, and (3) apoptosis (Figure 2C). Pathway cross-talk analysis demonstrated that the DFE may prevent the apoptosis of islet cells through suppressing the interleukin- (IL-) and interferon-induced inflammation/immune processes, which could activate downstream cell death signaling, including JAK-STAT/NF-κB/TNF pathways and others (Figures 3A and 3B). Additionally, the upregulated DEGs in the diabetes-DFE-VS-diabetes comparison were mainly enriched in the metabolic and insulin signaling pathways (Figure S4B). These results suggested that the DFE may decrease severe inflammation and immune response in the diabetic pancreas, which may thereby inhibit the islet cell apoptosis and increase insulin secretion (Figures 1E and 1F). Meanwhile, 22 upregulated and 11 downregulated DEMs displayed an opposite tendency between the DFE-VS-model and model-VS-control group comparisons (Figure 3C). Furthermore, some of these miRNAs have reportedly played vital roles in preventing the apoptosis of islet cells and facilitating the recovery of the pancreas functions. For example, *let-7d* could reduce insulin secretion and impair glucose tolerance;[15] here it was significantly upregulated in the diabetes-VS-normal comparison and downregulated with the DFE administration.

To investigate the transcriptional regulatory interactions underlying the DFE alleviating hyperglycemia in the pancreas, we constructed a miRNA-TF-gene regulatory network using both DEGs and DEMs with opposite expression trends between the two comparisons (diabetes-VS-normal and diabetes-DFE-VS-diabetes; Figure S5). This network contained 21 miRNAs, 22 TFs, and 124 genes, which consisted of 1,245 regulatory pairs. TFs of *Stat/Relb/NF-κB/Irf* families and *miR-148a/375/9a* regulating the inflammation and immune-related signaling pathways were represented as hub nodes, which may contribute to the hypoglycemic effect of DEF on DM (Figure 3D). For example, *miR-375* could target *Jak2* and act as an inhibitor to JAK/STAT signaling pathways,[16] and *miR-148a* is a repressor of IKBKB/NF-κB signaling, which inhibits the expression of inflammatory-related genes,[17] whereas *miR-9* works as an inflammation inhibitor through mediating the *TLR/NF-κB/mir-9* feedback loop and directly targeting the IL-1β/IRF/NF-κB/JAK/STAT pathway to positively regulate glucose-induced insulin secretion of β cells.[18] Combining the results of regulatory network analysis and functional enrichment of DEGs, we inferred that DFE alleviated hyperglycemia probably through inhibiting severe inflammation-induced islet cell apoptosis in the diabetic pancreas.

Transcriptome Profiling Revealed the DFE Effects on the Energy Metabolism and Lipid Accumulation in the Liver

The liver as a major target organ of insulin plays key roles in lipid metabolism, and fatty liver is a major risk factor of diabetes.[19] To reveal the potential molecular mechanisms of how DFE effects the diabetic liver, we investigated the transcriptome profiling in the liver as well. In the diabetes-VS-normal comparison, the expression levels of 1,222 genes (716 upregulated and 536 downregulated; Figure S6A) and 39 miRNAs (10 upregulated and 29 downregulated; Figure S6B) were significantly changed. The 716 upregulated DEGs were mainly enriched in the gluconeogenesis and non-alcoholic fatty liver disease (NAFLD) biological processes (Figure 4A), whereas the 536 downregulated DEGs were mainly associated with lipid metabolism, glutathione metabolism, and oxidoreductase activity and liver development processes (Figure 4A). Meanwhile, 418 DEGs were significantly upregulated, and 526 DEGs were downregulated in the diabetes-DFE-VS-diabetes comparison (Figure S6C). Notably, the DFE administration significantly restored multiple processes, which were dysregulated in the diabetic rats, such as gluconeogenesis, lipid metabolism, NAFLD, and oxidoreductase activity-related processes (Figure 4A). These results implied that DFE may improve the oxidoreductase activity and energy metabolism, and could decrease the lipid accumulation in the diabetic liver. For example, the expression levels of genes relevant to cholesterol and lipoprotein transport, such as *Apoa1-2/Apoc1-3/Lcn2/Rbp4*, showed reversed phase between the diabetes and diabetes-DFE groups (Figure 4B). Simultaneously, expression profiles of some miRNAs were markedly changed with DFE administration, such as *miR-375/miR-9a/miR-143/miR-127* and *miR-486/miR-451* (Figure 4C).

To investigate how the DEGs and DEMs were involved in the decrease of lipid with DFE administration, we constructed a miRNA-TF-gene regulatory network (219 nodes and 1,240 edges) using the DEGs and DEMs with opposite expression profiles in group comparisons of diabetes-DFE-VS-diabetes and diabetes-VS-normal (Figure S7). This regulatory network was involved in the energy and lipid metabolism, which may play important roles in decreasing lipid accumulation in the DFE group. Regulatory interactions between *miR-375/9a/143/127/192* and their targets appeared as core modules in our network, suggesting their key roles in the liver underlying the DFE alleviating diabetes (Figure 4D). For example, the *miR-192-Srebf1* axis plays important regulatory roles in hepatic steatosis, lipid accumulation, and the development of NAFLD.[20] The upregulation of miR-192 in the DFE-administrated rats may result in suppression to the expression of TF *Srebf1*, which could repress the expression of downstream genes related to the lipid biosynthesis, such as *Cyp7a1* and *Atp5g1*. TFs *Klf9/Klf13*, which were associated with hepatic steatosis and lipid accumulation,[21] were targeted by *miR-192/127/143* and acted as key nodes in the network to regulate the expressions of *Apoa1* and *Slc35c2*. The downregulation of

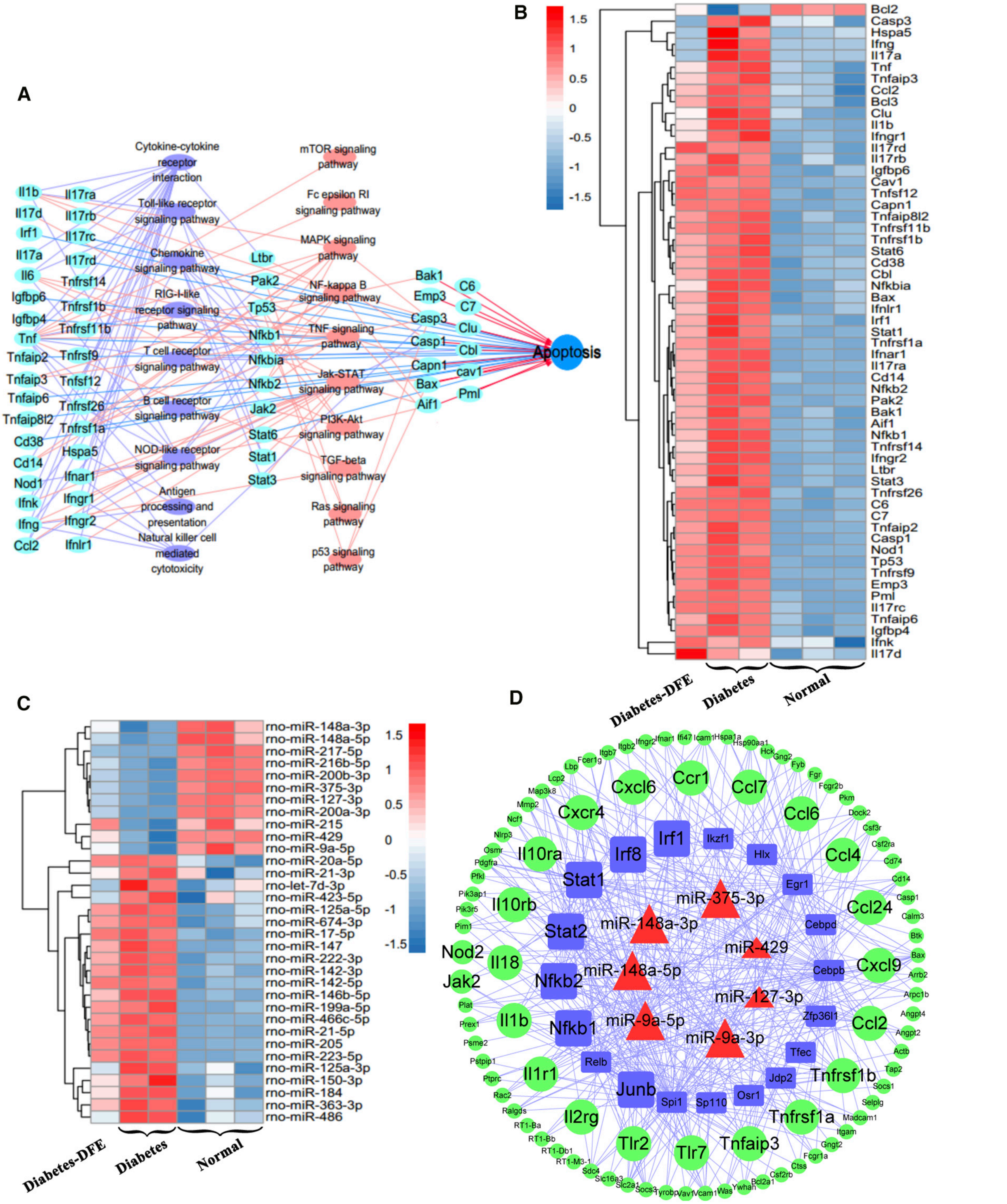

Figure 3. Functional and miRNA-TF-Targets Analysis of the Inflammation and Apoptosis-Related Genes in the Pancreas

(A) Crosstalk of genes involved in the inflammation and apoptosis processes. (B) Heatmap of the crosstalk genes that are involved in apoptosis. (C) Heatmap of differentially expressed miRNAs with opposite profiling in the comparisons of diabetes-DFE-VS-diabetes and diabetes-VS-normal. (D) Core miRNA-TF-genes regulatory network contributed to ameliorated DM under DFE treatment. Blue rectangles: TFs; red triangles: miRNAs; green circles: non-TF genes.

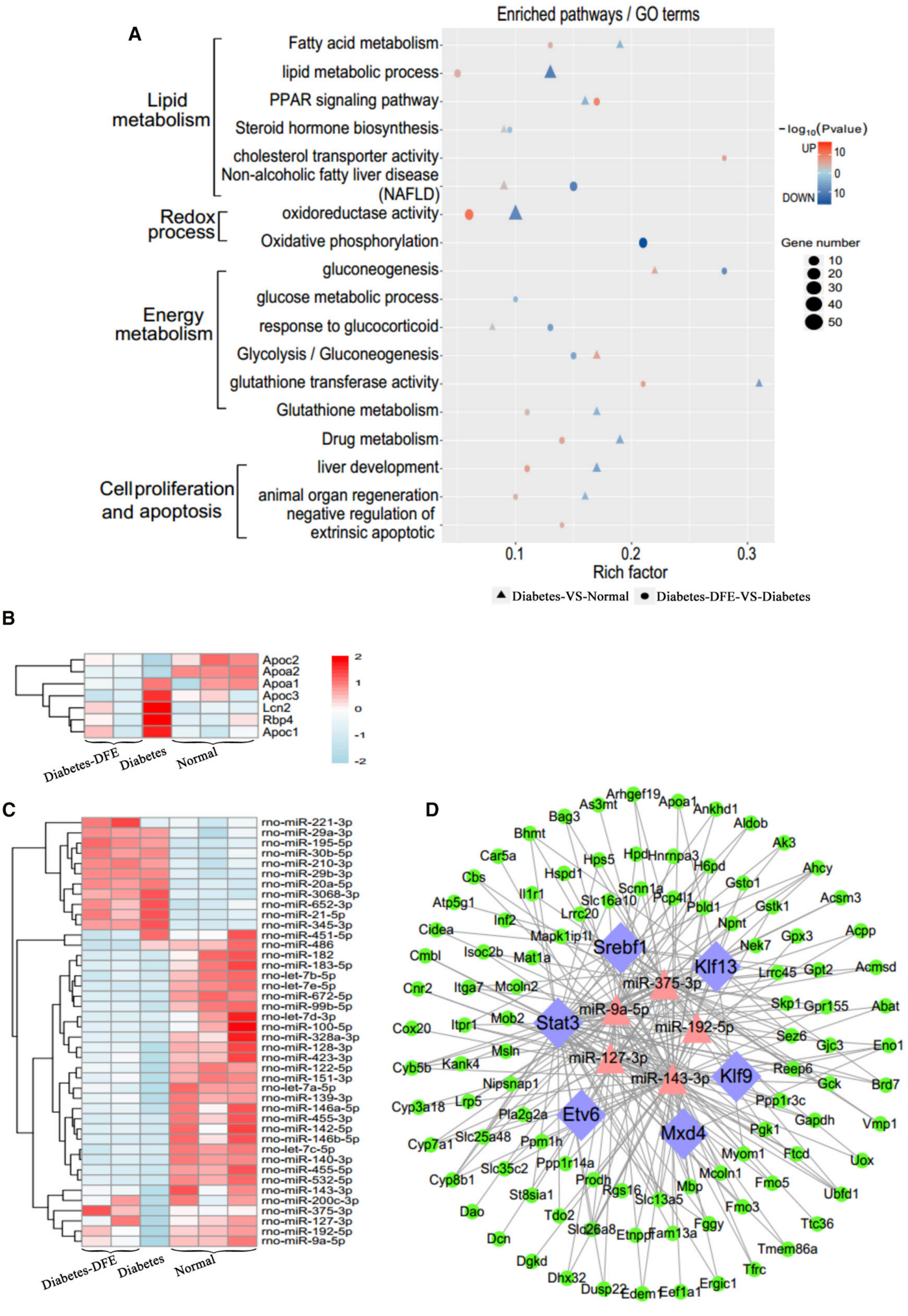

(legend on next page)

Apoa1 could bring about the reduced intake of plasma high-density lipoprotein cholesterol,[22] which thereby resulted in the decrease of lipid biosynthesis.

Experiments Validated that DFE Could Alleviate Hyperglycemia through Inhibiting Inflammation-Induced Islet Cell Apoptosis

To validate the anti-inflammation and anti-apoptosis effects of DFE on the diabetic pancreas predicted by the transcriptome profiling analysis, we performed cell and molecular biology experiments (Figure 5). Compared with the diabetes group, the levels of inflammatory markers, such as cytokines IL-1β and tumor necrosis factor alpha (TNF-α), were significantly decreased both in the serum and pancreas with DFE administration (Figure 5A), suggesting that DFE could alleviate severe inflammation in the diabetic pancreas. We also examined the cellular architecture and apoptosis of islet cells from the three groups. Tissue sections of the pancreas islets from normal and DFE groups showed well-preserved circular profiles, whereas pathological changes (massive destruction of the islets) were observed in the diabetic pancreas (Figure 5B). Meanwhile, terminal deoxynucleotidyl transferase (TdT)-mediated dUTP nick end labeling (TUNEL) assay demonstrated that DFE administration significantly decreased the islet cell apoptosis ratio (normal: ∼10%, diabetes: ∼82%, diabetes-DFE: 39%; Figure 5C). The expression levels of marker genes relevant to inflammation-induced apoptosis, such as *Casp3/Bcl2/Bax/Inf-γ/Tnf-α/Il-1β/Stat1/Il-17r*, evidenced that DFE could relieve severe inflammation and apoptosis in the diabetic pancreas as well (Figure 5D). Furthermore, the expression levels of insulin genes were significantly upregulated in the diabetes-DFE group compared with the diabetes group (Figure 5E), suggesting that DFE could inhibit the islet cell apoptosis and thereby increase the insulin level, which contributed to the prevention and treatment of diabetes.

Experiments Validated that DFE Alleviated Disordered Metabolism and Cell Apoptosis in the Diabetic Liver

The results of transcriptome profiling analysis indicated that DFE could decrease the gluconeogenesis, increase the oxidoreductase activity and lipid transport, partially attenuate NAFLD, and promote the regeneration of hepatocytes (Figure 6). To verify the effects of DFE on the diabetic liver, we measured the levels of some indicators related to the energy and lipid metabolisms, such as the total cholesterol (TC)/triglyceride (TG)/antioxidant capacity (TAC)/oxidative capacity, glycogen storage, and hepatocyte apoptosis ratio in the liver (Figure 6).

Consistent with the previous study, HFD and DEX could alter the glucose and lipid metabolism in the liver,[23] which resulted in lipid accumulation and lipotoxicity-induced cell death (Figure 6). Our results demonstrated that DFE administration significantly decreased the total cholesterol/triglyceride/oxidative capacity, whereas it increased the total antioxidant capacity and glycogen storage compared with the diabetes group (Figures 6A–6E). Meanwhile, the morphology and structure of hepatic cells were markedly changed with severe lipidosis in the diabetes group, whereas the DFE administration dramatically decreased lipid accumulation (Figure 6F). Furthermore, TUNEL experiments demonstrated the apoptosis of hepatocytes was significantly inhibited in the diabetes-DFE group compared with the diabetes group (apoptosis ratio: ∼72.9% in the diabetes group and ∼28.3% in the diabetes-DFE group; Figures 6G and 6H).

DISCUSSION

Diabetes mellitus is a metabolic disorder disease usually originating from the dysfunction of pancreatic β cells,[24] which could impair insulin target organs (e.g., the liver).[25] In this study, we assessed the anti-diabetic effect of DFE and explored the underlying mechanisms in the pancreas and liver tissues. Our data demonstrated: (1) DFE could reduce the severe inflammation in the diabetic pancreas and prevent the islet cell apoptosis, which could contribute to the protection or recovery of β cell function and mass; and (2) DFE could improve energy metabolism and strengthen the oxidoreductase activity and lipid transport in the diabetic liver, which may thereby decrease lipid accumulation and lipotoxicity-induced hepatocyte apoptosis.

The β cells make up 65%–80% of pancreas islet cells and are injury prone under excessive inflammation.[26] The low insulin and high glucose levels in diabetic rats (Figures 1E and 1F) may be caused by the loss of β cell mass (Figure 5C), which was consistent with a previous study.[27] Our data demonstrated that DFE administration could alleviate hyperglycemia through preventing β cell apoptosis via the anti-inflammation effect in the diabetic pancreas (Figures 5 and 7A). DFE administration significantly downregulated the expression level of a set of key genes (such as *Il-1β*, *Il-17r*, *Inf-γ*, *Tnf-a*, *Stat*, and *NF-κB*, etc.) compared with the diabetes group, suggesting that *Tnf-a*- and *Fas/FasL*-dependent apoptotic pathways may be inhibited in the islets with DFE administration[28,29] (Figure 5). Furthermore, DFE administration markedly increased the expression levels of genes involved in insulin signaling and metabolic pathways, including branch chain amino acids (BCAAs) degradation, fatty acid degradation, and PPAR signaling pathways (Figure S4B). The activation of PPAR signaling and fatty acid degradation pathways could reduce β cells apoptosis via upregulating fatty acid oxidation,[30] whereas increasing the BCAAs level could serve as a biomarker for impaired insulin action.[31] Additionally, miRNAs and TFs as important transcription regulators have participated in the genesis and development of diabetes.[32,33] The *miR-21/222/146* as pro-apoptosis agents[34] were

Figure 4. Transcriptome Profiling and Regulatory Network Analysis for the Livers in Three Groups

(A) Functional enrichment of DEGs in the diabetes-DFE-VS-diabetes and diabetes-VS-normal comparisons. (B) Heatmap for the gene expression of selected lipid transport genes. (C) Heatmap of differentially expressed miRNAs with opposite profiling in the comparisons of diabetes-DFE-VS-diabetes and diabetes-VS-normal. (D) Core miRNA-TF-genes regulatory network of differentially expressed miRNAs and genes in the comparison of diabetes-DFE-VS-diabetes from the liver. The legends of nodes are the same as in Figure 3.

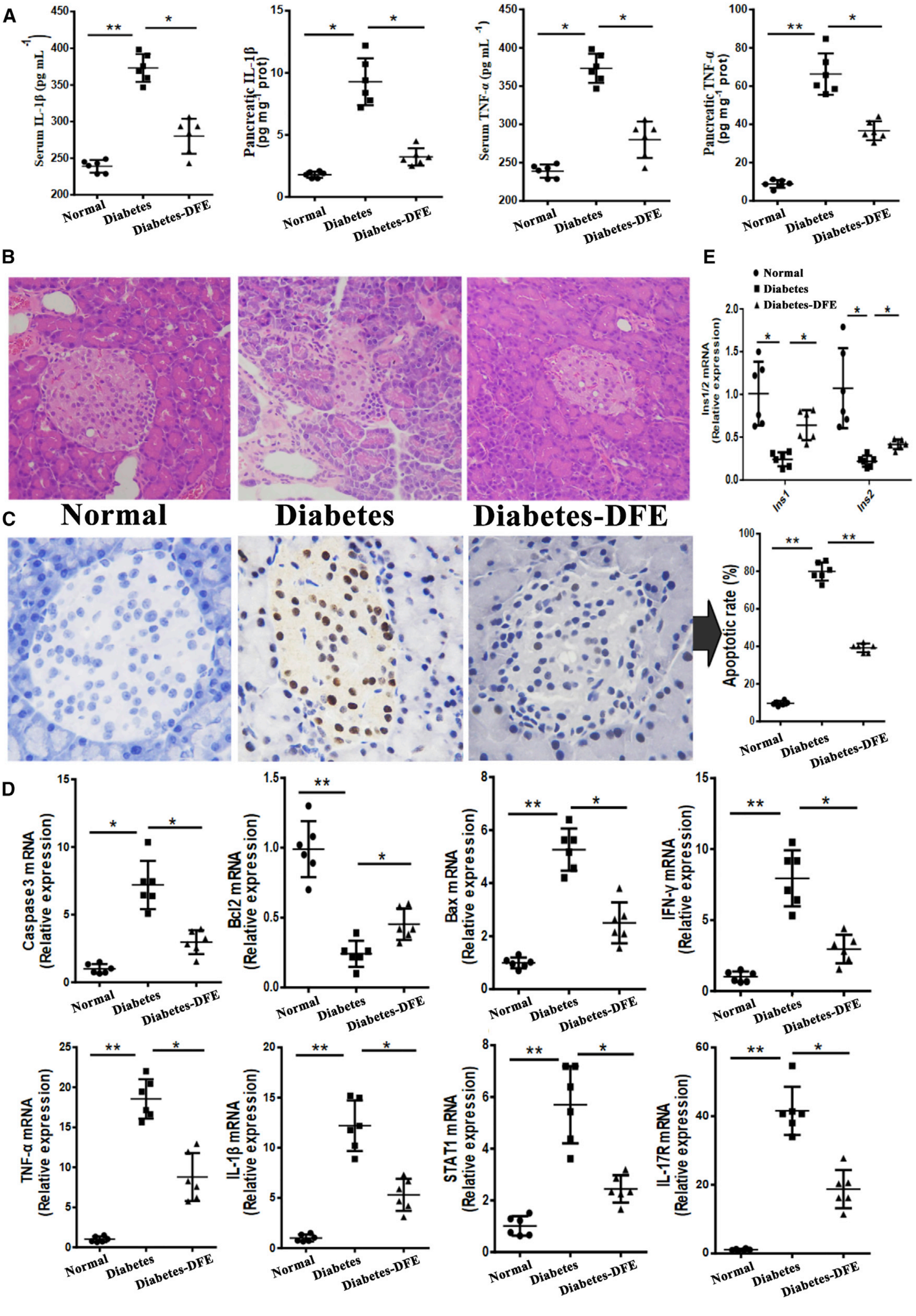

(legend on next page)

significantly downregulated in the DFE-VS-model comparison. The *miR-148a/375/9a* combined with TFs *Stat/Relb/NF-κBs/Irfs* acted as core modules regulating inflammation and immune-related signaling pathways, which may contribute to the alleviation of hyperglycemia in the diabetes-DFE group (Figure 3D). The miR-375 and miR-148 play important roles in maintaining pancreatic cell mass,[35] insulin biosynthesis, and normal glucose homeostasis,[36] and here they were highly expressed in the pancreas under DFE administration compared with the diabetes group (Figure 3C). Meanwhile, TFs *Stat/Relb/NF-κBs/Irfs* could promote cell apoptosis,[37,38] and here they were significantly downregulated with DFE administration (Figures 3C and 5D). Given the above findings together, we inferred that DFE played anti-inflammation and anti-apoptosis roles on the diabetic pancreas (Figure 7A), which could prevent the loss of β cell mass and recover the secretion of insulin (Figure 5).

The liver as a major target tissue of insulin maintains the homeostasis of metabolic processes. Lipid accumulation in hepatocytes is highly prevalent in diabetes and could increase the diabetes risk through disordered lipid metabolism and transport,[39,40] which may link the dysfunction of insulin to a metabolic disorder of the liver.[41] Excessive lipid accumulation may result in NAFLD (Figure 4A), and further triggers hepatocyte apoptosis and lowers cell regeneration.[42] Our results demonstrated that DFE could: (1) strengthen the oxidoreductase activity, lipid transport, and organ regeneration in diabetic livers (Figures 4A and 6); and (2) significantly decrease the glycogen consumption, lipid accumulation, and apoptosis in the hepatocytes (Figure 6). Genes (*Apo* family, *Lcn2*, and *Rbp4*, etc.) involved in lipid transport showed opposite expression profiles between the diabetes-DFE-VS-diabetes and diabetes-VS-normal comparisons (Figure 4B), suggesting their critical roles in decreasing lipid infiltration under DFE administration. Moreover, core regulatory modules with hubs *miR-9/375/127/143/192* and *Srebf1/Klfs* (Figure 4D) involved in the lipid metabolism and cell death may play important roles underlying the effects of DFE on the diabetic liver. For example, overexpressed *miR-9* could decrease intracellular lipid content in the liver,[43] and upregulating the expression of *miR-375* can inhibit mitochondrial autophagy in HCC cells,[44] which may contribute to the anti-apoptosis effect of DFE on the hepatocytes in our study (Figures 6G and 6H). Additionally, the *miR-192-Srebf1-Cyp7a1-Atp5g1* regulatory axis plays pivotal roles in the lipid metabolism/homeostasis.[20] The *miR-192/127/143* targeting TFs *Klf9/Klf13* regulate their downstream genes and here acted as key modules in our regulatory network of the liver (Figure 4D). These modules were associated with hepatic steatosis and lipid accumulation, and may inhibit the genesis and development of NAFLD. For example, *Slc35c2* as an Slc35 family member involved in the cell death process could enhance reactive oxygen species,[45] which was co-regulated by *mir-143* and *Klf13* in our network (Figure 4D), and its downregulation may contribute to the increase of antioxidant capacity (Figures 6C and 6D). Combining those findings, the most noteworthy effects of DFE on the diabetic liver may focus on maintaining the balance between the energy and lipid metabolism, and then ameliorating the side effects (NAFLD and hepatocyte apoptosis, etc.) induced by disordered gluconeogenesis and lipid accumulation (Figure 7B).

However, due to the RNA quality, the number of sequencing samples was not uniform in different groups or tissues. To avoid this limitation, we validated the key results of transcriptome profiling through physiological and molecular experiments. The high consistency and positive correlation between transcriptome profiling and experimental results indicated that our analyzed results from sequencing data reflected the real situation. Another limitation is that due to the complex compounds within TCM and possible interactions among those materials, the detailed biological active ingredients of TCM are often difficult to identify,[46] and the contribution of each compound on diabetes had not been investigated.

In summary, our findings establish the roles of DFE on regulating the β cell apoptosis and energy metabolism, suggesting that DFE has potential therapeutic implications for hyperglycemia and diabetes. Besides, transcriptome profiling analysis provides an effective approach to elucidate the regulatory pathways and mechanisms influenced by TCM intervention.[47] The combination of transcriptome profiling and regulatory network analyses could offer comprehensive views for changes of gene expression and underlying mechanisms,[48] which could provide new insights to explore the regulatory modules underlying the effects of TCM on diseases as well.

MATERIALS AND METHODS

Preparation of the DFE

The *D. fimbriatum* were obtained from Jiangxi Xiushui Miraculous Tea Industry (Jiangxi, China). DFE was extracted from dried stems of *D. fimbriatum*. In brief, the dried stem was pulverized and screened with a 100-mesh sieve and then was decocted with 50 vol (v/w) of boiling distilled water for 2 h. The filtrate was collected, and the residue was treated with the same procedure as above again. Finally, all the filtrates were put together and concentrated under a vacuum. The content of *D. fimbriatum* polysaccharides was measured by high-performance size exclusion chromatography (Figure S1), which accounted for 56.8% of the total exacts.

Animal Procedure and Sample Preparation

Male Sprague-Dawley rats weighing a range of 200–230 g and purchased from the Hubei Province Center for Disease Control and Prevention (China) were used in this study. Animal experiments were

Figure 5. Experimental Validation of the DFE Effects on Inflammation and Apoptosis Processes in the Pancreas

(A) The level of IL-1β and TNF-α in the serum/pancreas across the three groups. (B) Histopathological examination for pancreas of the three groups (original magnification ×200). (C) Terminal deoxynucleotidyl transferase-mediated dUTP nick end labeling (TUNEL) staining for the islets of the three groups (original magnification ×400). (D) Quantitative real-time PCR results for selected genes involved in the inflammation and apoptosis. (E) The relative expression levels of insulin genes among the three groups. All quantitative data are means ± SEM. $^{*}p < 0.05$ and $^{**}p < 0.01$ were determined by one-way ANOVA followed by Newman-Keuls post hoc tests.

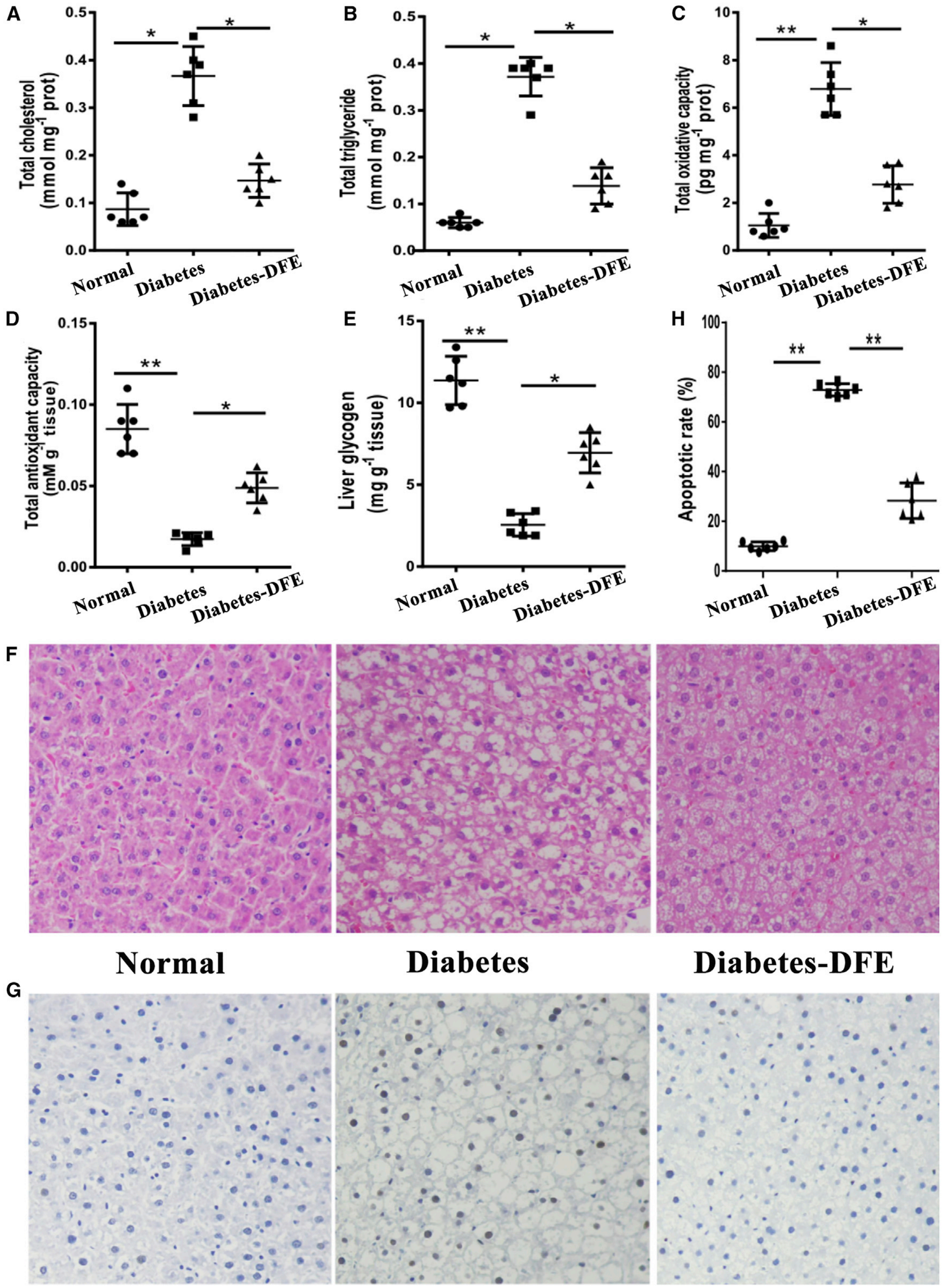

(legend on next page)

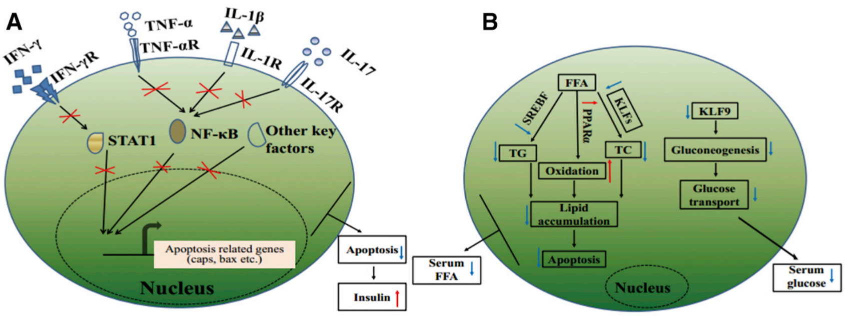

Figure 7. Potential Effects of DFE on the Diabetic Rats

(A) Potential model of the effects of DFE on the inflammation and apoptosis signaling pathways in the pancreas of diabetic rats. DFE may inhibit multiple signaling pathways, including IFN-γ, TNF-α, IL-1β, NF-κB, JAK-STAT, and IL-17 pathways. These signal transductions trigger several nucleus TFs, such as STAT and NF-κB, to regulate cell apoptosis. (B) Potential model showing the effects of DFE on alleviating lipid accumulation in the liver of diabetic rats.

approved and performed according to the guidelines of the Institutional Animal Care and Use Committee of HuaZhong University of Science and Technology (permission number: SCXK2015-0018). All reasonable efforts were made to minimize animal suffering.

Rats had free access to food (a standard chow diet) and water with controlled conditions (22°C ± 2°C, 12-h light-dark cycle). After an acclimation for 7 days, rats were randomly assigned to three groups: (1) non-diabetic group (n = 6, normal group): feed standard chow diet and saline throughout the whole experiment; (2) diabetes model group (n = 6, diabetes group): administrated with high-fat diet from 2 to 5 weeks and daily intraperitoneal injection with dexamethasone (DEX; 0.8 mg/kg) from the 4th to 5th week; and (3) DFE-treated group (n = 6, diabetes-DFE group): the same treatment as the diabetes group but additional DFE oral administration (100 mg/kg) throughout the experiment period (Figure 1A).

After experiments, rats were weighed and anesthetized with an intraperitoneal injection of sodium pentobarbital (1.5%, 0.2 mL/100 g). Blood samples (no anticoagulant) were centrifuged at 4,000 rpm/min for 10 min at 4°C. Aliquots of the supernatant were stored at −80°C for biochemical analysis. The liver and pancreas tissues were rapidly excised and treated in ice-cold PBS with liquid nitrogen. Total RNA with DNase treatment was isolated using the standard TRIzol protocol and quantified using a spectrophotometer (Nanodrop 2000c). Both the tissues and RNAs were stored at −80°C.

Library Preparation, Sequencing, and Data Processing

Libraries of RNA-seq (Ribo-Zero) and miRNA-seq were prepared and sequenced at BGI Bioinformatics Institute (Shenzhen, China). Library construction was on the basis of the TruSeq protocol (Illumina, USA), and the Illumina Hiseq2500 platform was employed to sequence these libraries using 2 × 150 bp pair-end strategy. The base-calling procedure was performed using the Illumina CASAVA v1.8.2 pipeline. All raw NGS data have been deposited in the Beijing Institute of Genomics Database (BIGD) (https://bigd.big.ac.cn/databases) under accession numbers BIGD: CRA000812 and CRA000813.

The adaptor removal and data filter procedures were performed using trimmomatic v.0.32 with default parameters.[49] RNA-seq reads with the length less than 35 bp after adaptor trimming or with poly(N) (>5 base) or low-quality bases (quality value ≤ 5, ratio of low quality base > 10%) were removed. For miRNA-seq data, reads with base N or unexpected length (>45 or <15 nt) or low quality (mean quality ≤ 20) were discarded. All of the downstream analyses were based on the clean data with high quality.

Abundance Estimation and Differential Expression Analysis for Genes and miRNAs

Clean reads from miRNA-seq were mapped to GenBank, Rfam, and Piwi to identify rRNA, tRNA, small nuclear RNA (snRNA), small nucleolar RNA (snoRNA), and PIWI-interacting RNA (piRNA). The unmapped reads above were aligned to canonical pre-miRNA sequences of rat from miRBase V21 to identify known miRNAs and generate expression profiles. The normalized values (transcripts per million reads [TPMs]) and read counts of expressed miRNAs in different samples were merged into matrix, respectively. The NOISeq were used to assess differentially expressed miRNAs across the three groups (false discovery rate [FDR] < 0.05 and |fold changes| > 1.5).

Transcript reassembly and quantification were processed according to the HISAT2-StringTie-ballgown pipeline[50] using Rattus norvegicus reference genome (version 6.0, known as "Rnor_6.0") and Ensembl v.83 annotation. The resulting transcripts were pooled across samples using the merge function of StringTie, discarding all contained and redundant isoforms. Furthermore, for a specific gene, we removed all the isoforms with a Jaccard distance score > 0.98 calculated by BEDTools2. The transcripts obtained above were used to build the "local" annotation file for further analysis. Normalized abundance of genes (fragments per kilobase of transcript per million

Figure 6. Experimental Validation of the Effects of DFE on the Liver

(A–D) The concentrations of total cholesterol (A), triglyceride (B), oxidant capacity (C), and antioxidative capacity (D) in the liver across the three groups. (E) The glycogen storage in the liver. (F) Histopathological examination for the liver of the three groups. (G) TUNEL staining for the hepatocyte among three groups. (H) Digital display for the apoptosis ratio. All quantitative data are means ± SEM. *p < 0.05 and **p < 0.01 were determined by one-way ANOVA followed by Newman-Keuls post hoc tests.

fragments mapped [FPKM]) were estimated using StringTie with the local annotation. Genes with FPKM > 1 in any condition (average across replicates) were kept for further analysis. The DEG analysis was performed using ballgown and NOISeq with default parameters and the significance threshold (FDR < 0.05 and |fold change| > 4).

Biochemical Analyses

Blood samples from tail veins were used to determine serum glucose concentrations by glucometer (Roche, Switzerland). Overnight-fasted rats were given glucose by oral gavage (2.5 g/kg), and blood samples were collected at 0 (before glucose administration), 30, 60, and 120 min after glucose administration. Insulin concentration was measured by ELISA kit (DRG, Germany). The levels of IL-1β and TNF-α were determined by ELISA kit (Cloud Clone, Wuhan, China) according to the manufacturer's recommendations. The total cholesterol (TC), triglyceride (TG), antioxidant capacity (TAC), and oxidant capacity (TOC) were detected by kits (Jiancheng Bioengineering, China).

Histopathological Examination and Terminal Deoxynucleotidyl Transferase-Mediated dUTP Nick End Labeling (TUNEL) Staining

Tissues were dehydrated in graded series of alcohol, embedded in paraffin, and sectioned in 5-mm thickness by using a microtome (ARM-3600; Histo-Line Laboratories, Italy). The sections were dewaxed in three changes of xylene, hydrated in two changes of 100% ethanol, followed by 95% and 80% ethanol, rinsed with water, and then stained with H&E. Histopathological examinations were carried out under light microscope with attached photograph machine (Nikon H600L, Japan).

Apoptotic cells were visualized by TUNEL staining according to the manufacturer's manual. Tissue sections were digested with Proteinase K and kept for 15 min at 37°C. Slides were placed in a decolorization bed and washed three times (5 min for each time), and then were incubated with terminal deoxynucleotidyl transferase (TdT) buffer followed by TdT reaction solution containing TdT and dUTP for 60 min at 37°C. Further, slides were washed with PBS for 5 min and incubated with anti-digoxigenin peroxidase for 30 min. Color was developed using 0.05% diaminobenzidine (DAB). Sections were then washed, dehydrated, and mounted. Apoptotic cells were identified by a brown stain over the nuclei.

Quantitative Real-Time RT-PCR

cDNA was synthesized using PrimeScript RT reagent Kit with gDNA Eraser (QIAGEN, Germany); then real-time PCR was performed using SYBR Premix Ex TaqTMII (Takara Biotechnology, China) in a Bio-Rad C1000 detecting system (Bio-Rad, USA). β-Actin was used as the control, and the fold change for all samples was calculated by the $2^{-\Delta\Delta Ct}$ method.

Statistical and Regulatory Network Analysis

SPSS v.17.0 was used for the biochemical analysis. Data from replicate samples are shown as mean ± SEM. Datasets with more than two groups were assessed by one-way ANOVA followed by Newman-Keuls post hoc tests, and $p < 0.05$ was considered the statistically significant threshold. Kyoto Encyclopedia of Genes and Genomes (KEGG) and Gene Ontology (GO) enrichment analyses were performed on DAVID. The gene list of TFs was downloaded from AnimalTFDB. The method used for regulatory network analysis was described in our previous work.[51] Graphs were created in Cytoscape and R studio.

Ethics Statement

Animal experiments were approved and performed according to the guidelines of Institutional Animal Care and Use Committee of Hua Zhong University of Science and Technology.

SUPPLEMENTAL INFORMATION

Supplemental Information can be found online at https://doi.org/10.1016/j.omtn.2019.10.033.

AUTHOR CONTRIBUTIONS

Y.Z., X.Y., and A.-Y.G. designed this work. Q.Z. performed the whole bioinformatics analysis and wrote the manuscript. J.L. performed the experiments and helped edit the manuscript. Q.Z., M.L., and G.-Y.X. performed the data visualization. W.Z. and Y.W. assisted the experiments. Q.Z., Y.Z., and A.-Y.G. revised the manuscript.

CONFLICTS OF INTEREST

The authors declare no competing interests.

ACKNOWLEDGMENTS

We are grateful to Qi Wang for his good suggestions on the histopathological examination and TUNEL experiments. We thank Chunjie Liu for his useful comments on the manuscript. This work was supported by the National Natural Science Foundation of China (NSFC) (grants 31801113, 31822030, 31771458, and 81573013); China Postdoctoral Science Foundation (grant 2018M632830); National Basic Research Program of China (grant 2015CB931802); PCSIRT (grant IRT13016); and Open Project funded by Key Laboratory of Carcinogenesis and Translational Research (Ministry of Education, Beijing, 2017 Open Project-6).

REFERENCES

1. American Diabetes Association (2014). Diagnosis and classification of diabetes mellitus. Diabetes Care *37* (*Suppl 1*), S81–S90.
2. Schofield, J.D., Liu, Y., Rao-Balakrishna, P., Malik, R.A., and Soran, H. (2016). Diabetes Dyslipidemia. Diabetes Ther. *7*, 203–219.
3. Stein, S.A., Lamos, E.M., and Davis, S.N. (2013). A review of the efficacy and safety of oral antidiabetic drugs. Expert Opin. Drug Saf. *12*, 153–175.
4. Li, W.L., Zheng, H.C., Bukuru, J., and De Kimpe, N. (2004). Natural medicines used in the traditional Chinese medical system for therapy of diabetes mellitus. J. Ethnopharmacol. *92*, 1–21.
5. Tong, X.L., Wu, S.T., Lian, F.M., Zhao, M., Zhou, S.P., Chen, X.Y., Yu, B., Zhen, Z., Qi, L.W., Li, P., et al. (2013). The safety and effectiveness of TM81, a Chinese herbal medicine, in the treatment of type 2 diabetes: a randomized double-blind placebo-controlled trial. Diabetes Obes. Metab. *15*, 448–454.
6. Lian, F., Tian, J., Chen, X., Li, Z., Piao, C., Guo, J., Ma, L., Zhao, L., Xia, C., Wang, C.Z., et al. (2015). The Efficacy and Safety of Chinese Herbal Medicine Jinlida as Add-On Medication in Type 2 Diabetes Patients Ineffectively Managed by Metformin

Monotherapy: A Double-Blind, Randomized, Placebo-Controlled, Multicenter Trial. PLoS ONE *10*, e0130550.

7. Lee, S.W., Nam, M.H., and Lee, B.C. (2017). Herbal acupuncture for type 2 diabetes: A meta-analysis. Exp. Ther. Med. *13*, 3249–3256.
8. Tang, H., Zhao, T., Sheng, Y., Zheng, T., Fu, L., and Zhang, Y. (2017). Dendrobium officinale Kimura et Migo: A Review on Its Ethnopharmacology, Phytochemistry, Pharmacology, and Industrialization. Evid. Based Complement Alternat. Med. 2017, 7436259.
9. Lv, G.Y., Yan, M.Q., and Chen, S.H. (2013). [Review of pharmacological activities of Dendrobium officinale based on traditional functions]. Zhongguo Zhongyao Zazhi *38*, 489–493.
10. Li, T.M., and Deng, M.Z. (2012). [Effect of dendrobium mixture on hypoglycemic and the apoptosis of islet in rats with type 2 diabetic mellitus]. Zhong Yao Cai *35*, 765–769.
11. Chang, H.L. (2015). Effects of Dendrobium Candidum on Phosphorylation Expression of JNK and AKT Protein in Rats with Type 2 Diabetes Mellitus. Chin. Pharm. Aff. *29*, 54–57.
12. Qian, X., Li, X., Ilori, T.O., Klein, J.D., Hughey, R.P., Li, C.J., Alli, A.A., Guo, Z., Yu, P., Song, X., and Chen, G. (2015). RNA-seq analysis of glycosylation related gene expression in STZ-induced diabetic rat kidney inner medulla. Front. Physiol. *6*, 274.
13. Cnop, M., Abdulkarim, B., Bottu, G., Cunha, D.A., Igoillo-Esteve, M., Masini, M., Turatsinze, J.V., Griebel, T., Villate, O., Santin, I., et al. (2014). RNA sequencing identifies dysregulation of the human pancreatic islet transcriptome by the saturated fatty acid palmitate. Diabetes *63*, 1978–1993.
14. Kurtz, C.L., Peck, B.C.E., Fannin, E.E., Beysen, C., Miao, J., Landstreet, S.R., Ding, S., Turaga, V., Lund, P.K., Turner, S., et al. (2014). MicroRNA-29 fine-tunes the expression of key FOXA2-activated lipid metabolism genes and is dysregulated in animal models of insulin resistance and diabetes. Diabetes *63*, 3141–3148.
15. Frost, R.J.A., and Olson, E.N. (2011). Control of glucose homeostasis and insulin sensitivity by the Let-7 family of microRNAs. Proc. Natl. Acad. Sci. USA *108*, 21075–21080.
16. Yin, L.H., Zheng, X.Q., Li, H.Y., Bi, L.X., Shi, Y.F., Ye, A.F., Wu, J.B., and Gao, S.M. (2015). Epigenetic deregulated miR-375 contributes to the constitutive activation of JAK2/STAT signaling in myeloproliferative neoplasm. Leuk. Res. *39*, 471–478.
17. Patel, V., Carrion, K., Hollands, A., Hinton, A., Gallegos, T., Dyo, J., Sasik, R., Leire, E., Hardiman, G., Mohamed, S.A., et al. (2015). The stretch responsive microRNA miR-148a-3p is a novel repressor of IKBKB, NF-κB signaling, and inflammatory gene expression in human aortic valve cells. FASEB J. *29*, 1859–1868.
18. Wang, Y., Han, Z., Fan, Y., Zhang, J., Chen, K., Gao, L., Zeng, H., Cao, J., and Wang, C. (2017). MicroRNA-9 Inhibits NLRP3 Inflammasome Activation in Human Atherosclerosis Inflammation Cell Models through the JAK1/STAT Signaling Pathway. Cell. Physiol. Biochem. *41*, 1555–1571.
19. Loria, P., Lonardo, A., and Anania, F. (2013). Liver and diabetes. A vicious circle. Hepatol. Res *43*, 51–64.
20. Lin, Y., Ding, D., Huang, Q., Liu, Q., Lu, H., Lu, Y., Chi, Y., Sun, X., Ye, G., Zhu, H., et al. (2017). Downregulation of miR-192 causes hepatic steatosis and lipid accumulation by inducing SREBF1: Novel mechanism for bisphenol A-triggered non-alcoholic fatty liver disease. Biochim. Biophys. Acta Mol. Cell Biol. Lipids *1862*, 869–882.
21. Wu, Z., and Wang, S. (2013). Role of kruppel-like transcription factors in adipogenesis. Dev. Biol. *373*, 235–243.
22. Neuschwander-Tetri, B.A., Loomba, R., Sanyal, A.J., Lavine, J.E., Van Natta, M.L., Abdelmalek, M.F., Chalasani, N., Dasarathy, S., Diehl, A.M., Hameed, B., et al.; NASH Clinical Research Network (2015). Farnesoid X nuclear receptor ligand obeticholic acid for non-cirrhotic, non-alcoholic steatohepatitis (FLINT): a multicentre, randomised, placebo-controlled trial. Lancet *385*, 956–965.
23. Harasim-Symbor, E., Konstantynowicz-Nowicka, K., and Chabowski, A. (2016). Additive effects of dexamethasone and palmitate on hepatic lipid accumulation and secretion. J. Mol. Endocrinol. *57*, 261–273.
24. Ashcroft, F.M., and Rorsman, P. (2012). Diabetes mellitus and the β cell: the last ten years. Cell *148*, 1160–1171.
25. Michael, M.D., Kulkarni, R.N., Postic, C., Previs, S.F., Shulman, G.I., Magnuson, M.A., and Kahn, C.R. (2000). Loss of insulin signaling in hepatocytes leads to severe insulin resistance and progressive hepatic dysfunction. Mol. Cell *6*, 87–97.
26. Montane, J., Cadavez, L., and Novials, A. (2014). Stress and the inflammatory process: a major cause of pancreatic cell death in type 2 diabetes. Diabetes Metab. Syndr. Obes. *7*, 25–34.
27. Thomsen, S.K., and Gloyn, A.L. (2014). The pancreatic β cell: recent insights from human genetics. Trends Endocrinol. Metab. *25*, 425–434.
28. Suk, K., Kim, S., Kim, Y.H., Kim, K.A., Chang, I., Yagita, H., Shong, M., and Lee, M.S. (2001). IFN-gamma/TNF-alpha synergism as the final effector in autoimmune diabetes: a key role for STAT1/IFN regulatory factor-1 pathway in pancreatic beta cell death. J. Immunol. *166*, 4481–4489.
29. Kutlu, B., Cardozo, A.K., Darville, M.I., Kruhøffer, M., Magnusson, N., Ørntoft, T., and Eizirik, D.L. (2003). Discovery of gene networks regulating cytokine-induced dysfunction and apoptosis in insulin-producing INS-1 cells. Diabetes *52*, 2701–2719.
30. Yang, H., and Li, X. (2012). The role of fatty acid metabolism and lipotoxicity in pancreatic β-cell injury: Identification of potential therapeutic targets. Acta Pharm. Sin. B *2*, 396–402.
31. Long, W., Saffer, L., Wei, L., and Barrett, E.J. (2000). Amino acids regulate skeletal muscle PHAS-I and p70 S6-kinase phosphorylation independently of insulin. Am. J. Physiol. Endocrinol. Metab. *279*, E301–E306.
32. Lovis, P., Roggli, E., Laybutt, D.R., Gattesco, S., Yang, J.Y., Widmann, C., Abderrahmani, A., and Regazzi, R. (2008). Alterations in microRNA expression contribute to fatty acid-induced pancreatic beta-cell dysfunction. Diabetes *57*, 2728–2736.
33. Guo, S., Dai, C., Guo, M., Taylor, B., Harmon, J.S., Sander, M., Robertson, R.P., Powers, A.C., and Stein, R. (2013). Inactivation of specific β cell transcription factors in type 2 diabetes. J. Clin. Invest. *123*, 3305–3316.
34. Chen, H., Lan, H.Y., Roukos, D.H., and Cho, W.C. (2014). Application of microRNAs in diabetes mellitus. J. Endocrinol. *222*, R1–R10.
35. Poy, M.N., Hausser, J., Trajkovski, M., Braun, M., Collins, S., Rorsman, P., Zavolan, M., and Stoffel, M. (2009). miR-375 maintains normal pancreatic alpha- and beta-cell mass. Proc. Natl. Acad. Sci. USA *106*, 5813–5818.
36. Melkman-Zehavi, T., Oren, R., Kredo-Russo, S., Shapira, T., Mandelbaum, A.D., Rivkin, N., Nir, T., Lennox, K.A., Behlke, M.A., Dor, Y., and Hornstein, E. (2011). miRNAs control insulin content in pancreatic β-cells via downregulation of transcriptional repressors. EMBO J. *30*, 835–845.
37. Silverman, N., and Maniatis, T. (2001). NF-kappaB signaling pathways in mammalian and insect innate immunity. Genes Dev. *15*, 2321–2342.
38. Mankan, A.K., Lawless, M.W., Gray, S.G., Kelleher, D., and McManus, R. (2009). NF-kappaB regulation: the nuclear response. J. Cell. Mol. Med. *13*, 631–643.
39. Brenner, R.R., Ayala, S., and Garda, H.A. (2001). Effect of dexamethasone on the fatty acid composition of total liver microsomal lipids and phosphatidylcholine molecular species. Lipids *36*, 1337–1345.
40. Hazlehurst, J.M., Woods, C., Marjot, T., Cobbold, J.F., and Tomlinson, J.W. (2016). Non-alcoholic fatty liver disease and diabetes. Metabolism *65*, 1096–1108.
41. Bechmann, L.P., Hannivoort, R.A., Gerken, G., Hotamisligil, G.S., Trauner, M., and Canbay, A. (2012). The interaction of hepatic lipid and glucose metabolism in liver diseases. J. Hepatol. *56*, 952–964.
42. Alkhouri, N., Carter-Kent, C., and Feldstein, A.E. (2011). Apoptosis in nonalcoholic fatty liver disease: diagnostic and therapeutic implications. Expert Rev. Gastroenterol. Hepatol. *5*, 201–212.
43. Ao, R., Wang, Y., Tong, J., and Wang, B.F. (2016). Altered microRNA-9 Expression Level is Directly Correlated with Pathogenesis of Nonalcoholic Fatty Liver Disease by Targeting Onecut2 and SIRT1. Med. Sci. Monit. *22*, 3804–3819.
44. Chang, Y., Yan, W., He, X., Zhang, L., Li, C., Huang, H., Nace, G., Geller, D.A., Lin, J., and Tsung, A. (2012). miR-375 inhibits autophagy and reduces viability of hepatocellular carcinoma cells under hypoxic conditions. Gastroenterology *143*, 177–187.e8.
45. Baines, C.P., and Molkentin, J.D. (2009). Adenine nucleotide translocase-1 induces cardiomyocyte death through upregulation of the pro-apoptotic protein Bax. J. Mol. Cell. Cardiol. *46*, 969–977.

46. Fung, F.Y., and Linn, Y.C. (2015). Developing traditional chinese medicine in the era of evidence-based medicine: current evidences and challenges. Evid. Based Complement Alternat. Med *2015*, 425037.

47. Gu, P., and Chen, H. (2014). Modern bioinformatics meets traditional Chinese medicine. Brief. Bioinform. *15*, 984–1003.

48. Zhang, H.M., Li, Q., Zhu, X., Liu, W., Hu, H., Liu, T., Cheng, F., You, Y., Zhong, Z., Zou, P., et al. (2016). miR-146b-5p within BCR-ABL1-Positive Microvesicles Promotes Leukemic Transformation of Hematopoietic Cells. Cancer Res. *76*, 2901–2911.

49. Bolger, A.M., Lohse, M., and Usadel, B. (2014). Trimmomatic: a flexible trimmer for Illumina sequence data. Bioinformatics *30*, 2114–2120.

50. Pertea, M., Kim, D., Pertea, G.M., Leek, J.T., and Salzberg, S.L. (2016). Transcript-level expression analysis of RNA-seq experiments with HISAT, StringTie and Ballgown. Nat. Protoc. *11*, 1650–1667.

51. Lin, Y., Zhang, Q., Zhang, H.M., Liu, W., Liu, C.J., Li, Q., and Guo, A.Y. (2015). Transcription factor and miRNA co-regulatory network reveals shared and specific regulators in the development of B cell and T cell. Sci. Rep. *5*, 15215.

OMTN, Volume *19*

Supplemental Information

Systematic Transcriptome and Regulatory Network Analyses Reveal the Hypoglycemic Mechanism of *Dendrobium fimbriatum*

Qiong Zhang, Jing Li, Mei Luo, Gui-Yan Xie, Weiwei Zeng, Yuxin Wu, Yanhong Zhu, Xiangliang Yang, and An-Yuan Guo

Supplementary Figures and Tables

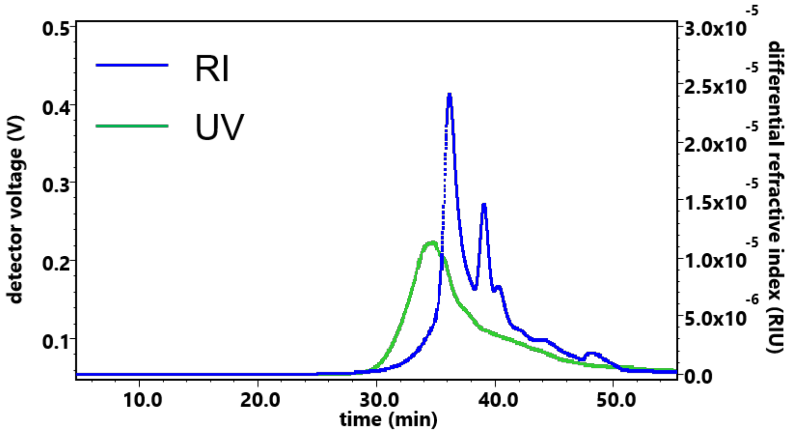

Supplementary Figure 1 | High performance size exclusion chromatography (HPSEC)/refractive index (RI) of *D. fimbriatum.*

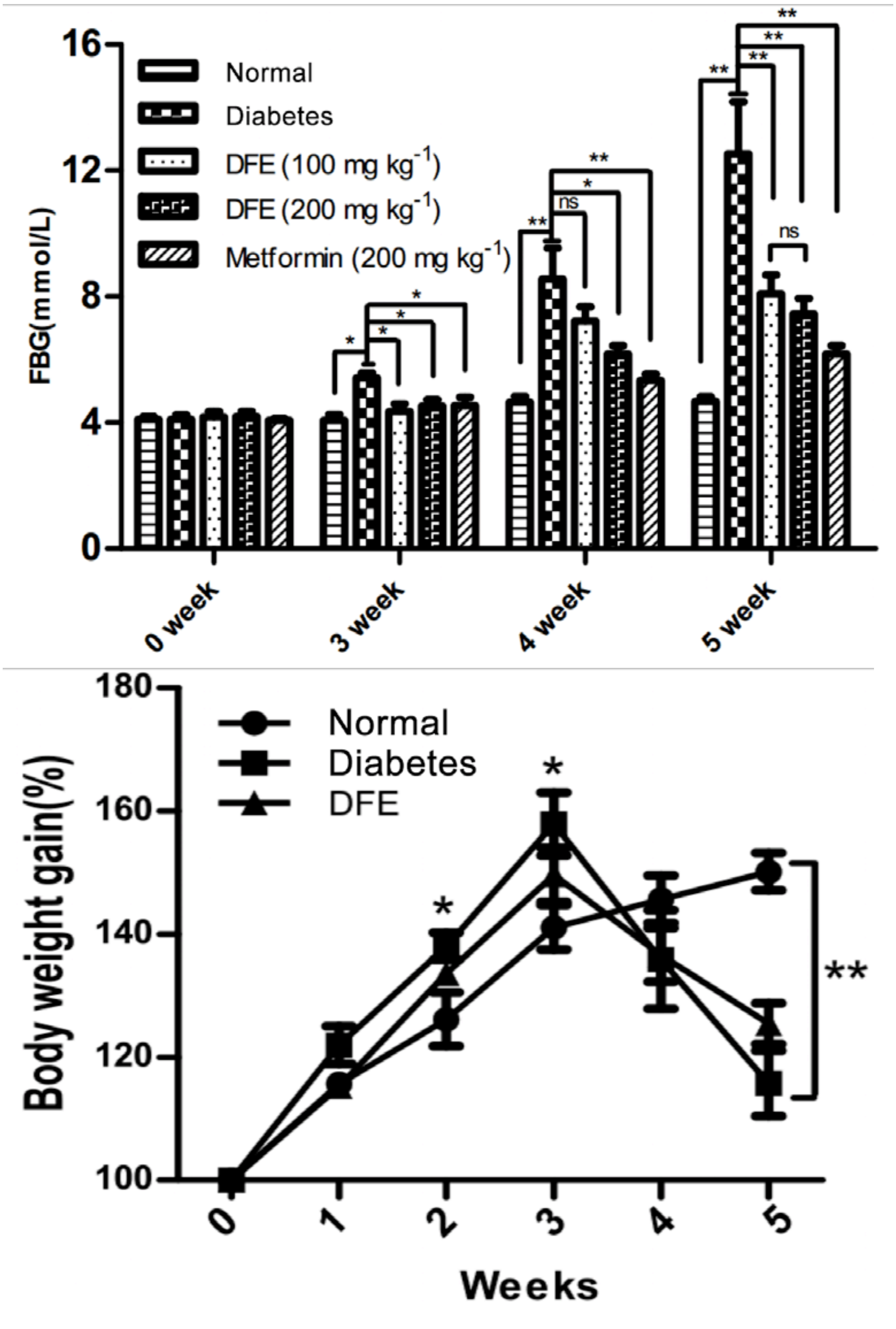

Supplementary Figure 2 | The dose effect of DFE alleviating diabetes symptom and body weight gain on diabetes rats. All quantitative data are reported as means ± SEM. *P-value < 0.05 and **P-value < 0.01 were determined by one-way ANOVA followed by Newman–Keuls post hoc tests. The ns represents no-significance.

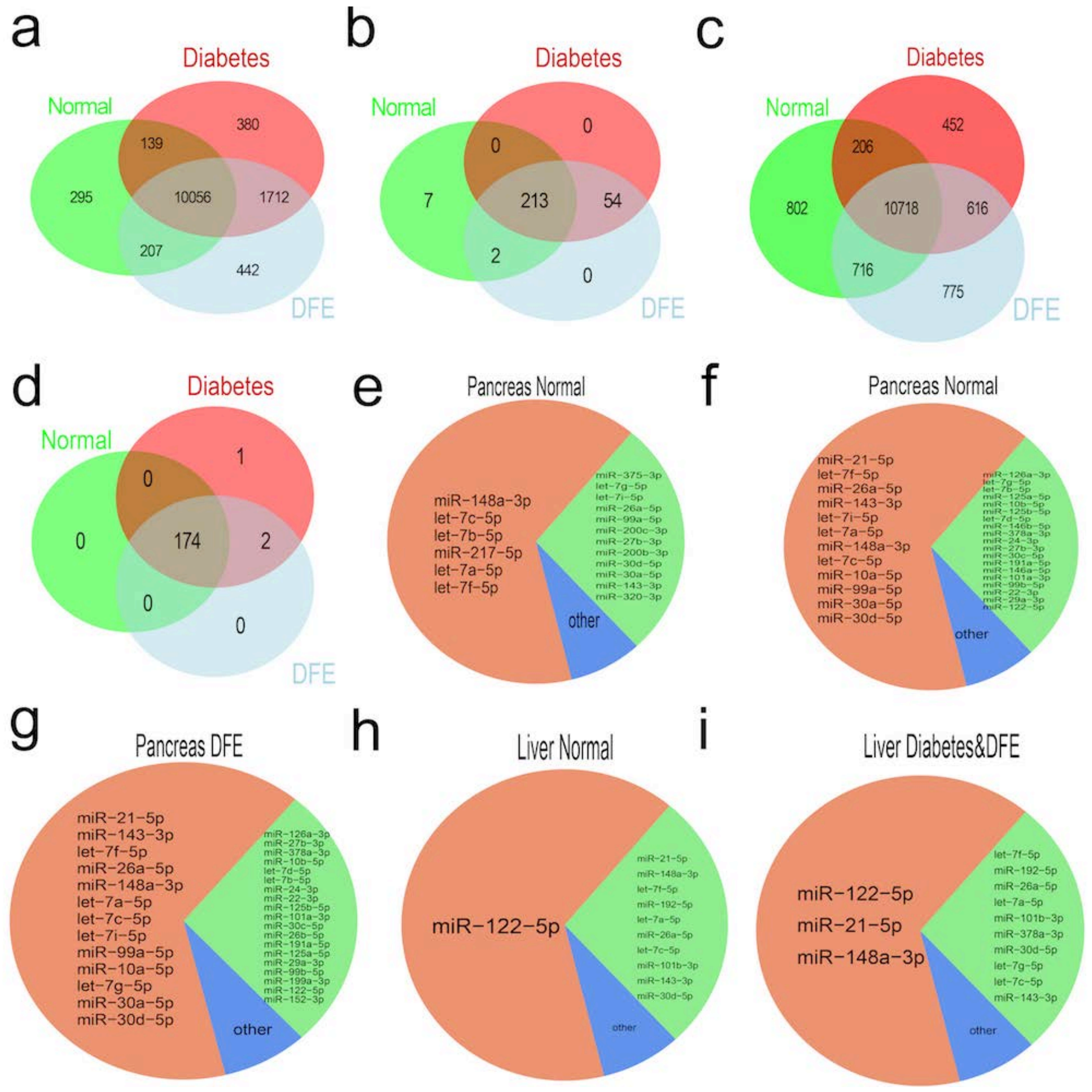

Supplementary Figure 3 | Transcriptome profiles of RNA-Seq and miRNA-Seq in the pancreas and liver across the normal, diabetes and diabetes-DFE groups. (A) Genes with FPKM>1 in the pancreas among the three groups; (B) miRNAs with TPM>10 in the pancreas among the three groups; (C) Genes with FPKM>1 in the liver among the three groups; (D) MiRNAs with TPM>10 in the liver among the three groups; (E-G) Component percentage of expressed miRNAs in the pancreas of the three groups, yellow: 70%, green: 20%, blue: 10%; (H, I) Component percentage of expressed miRNAs in the liver of the three groups.

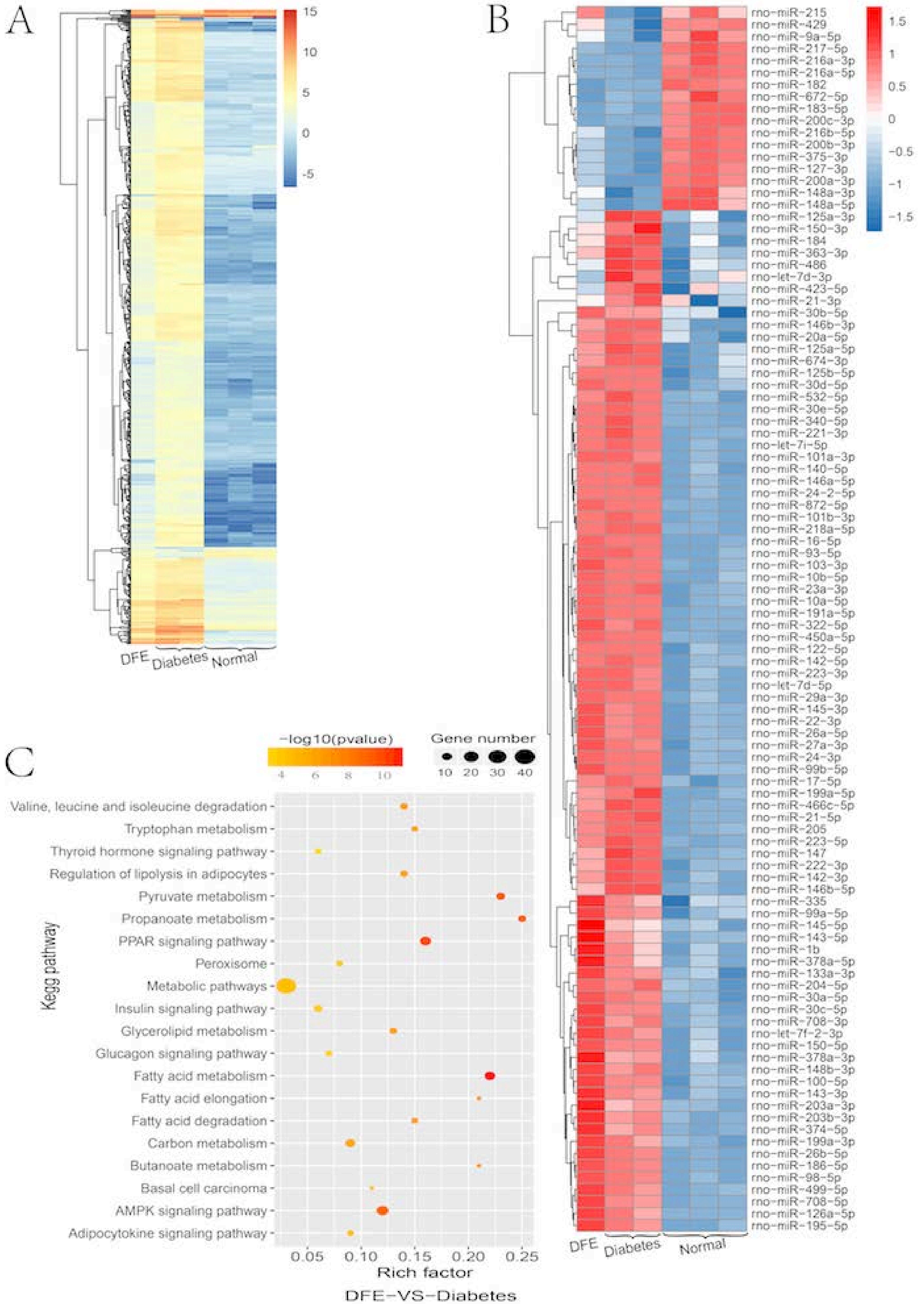

Supplementary Figure 4 | Heatmap of differentially expressed miRNAs in the pancreas and KEGG enrichment result of up-regulated DEGs in the comparison of diabetes-DFE-VS-diabetes. (A) Heatmap for 588 DEGs with opposite expression profiles in the comparisons of diabetes-DFE-VS-diabetes and diabetes -VS-normal; (B) Heatmap of DEMs in the pancreas among the three groups; (C) Top 20 terms of KEGG enrichment results in the comparison of diabetes-DFE-VS-diabetes.

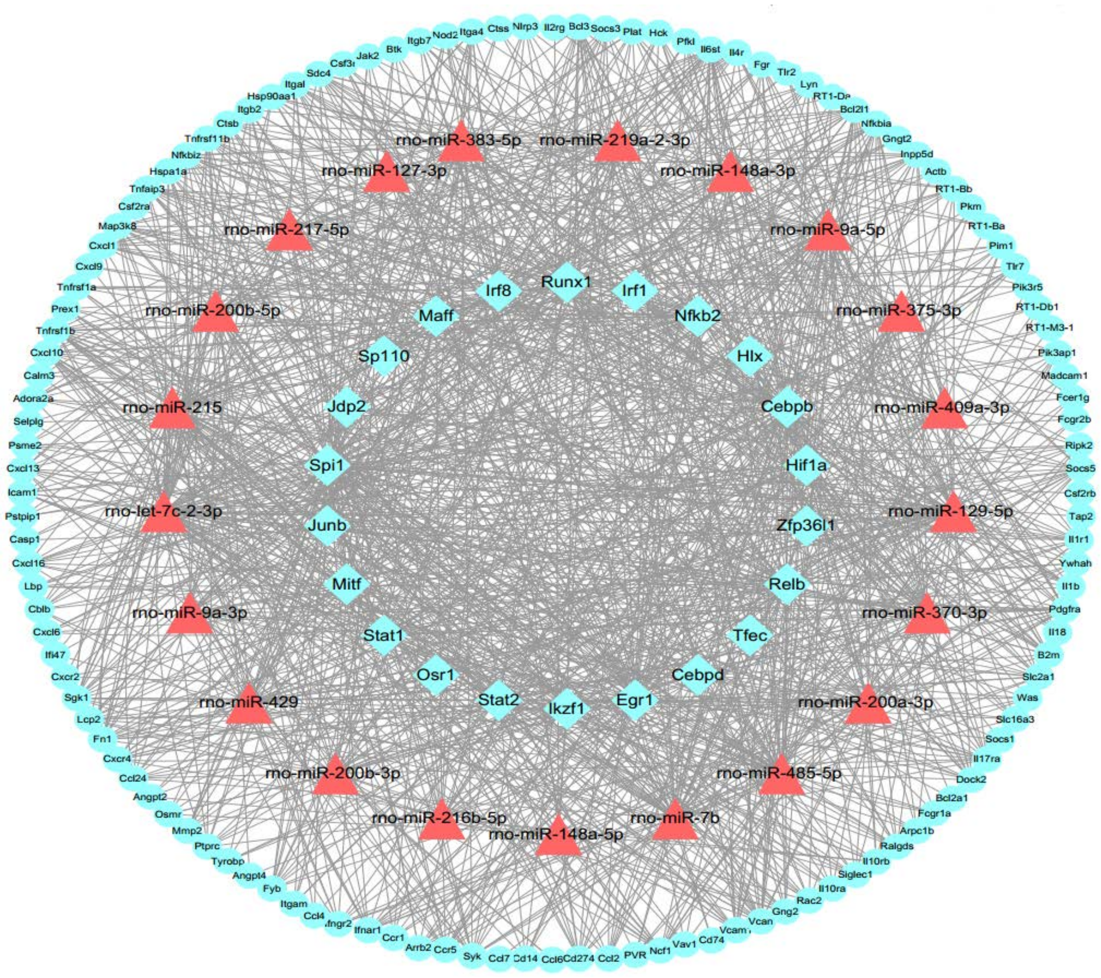

Supplementary Figure 5 | miRNAs-TFs-genes regulatory network involved in the effects of DFE on alleviating inflammation and apoptosis in the diabetic pancreas. Blue rectangles: TFs; red triangles: miRNAs; blue cycles: NON-TF genes.

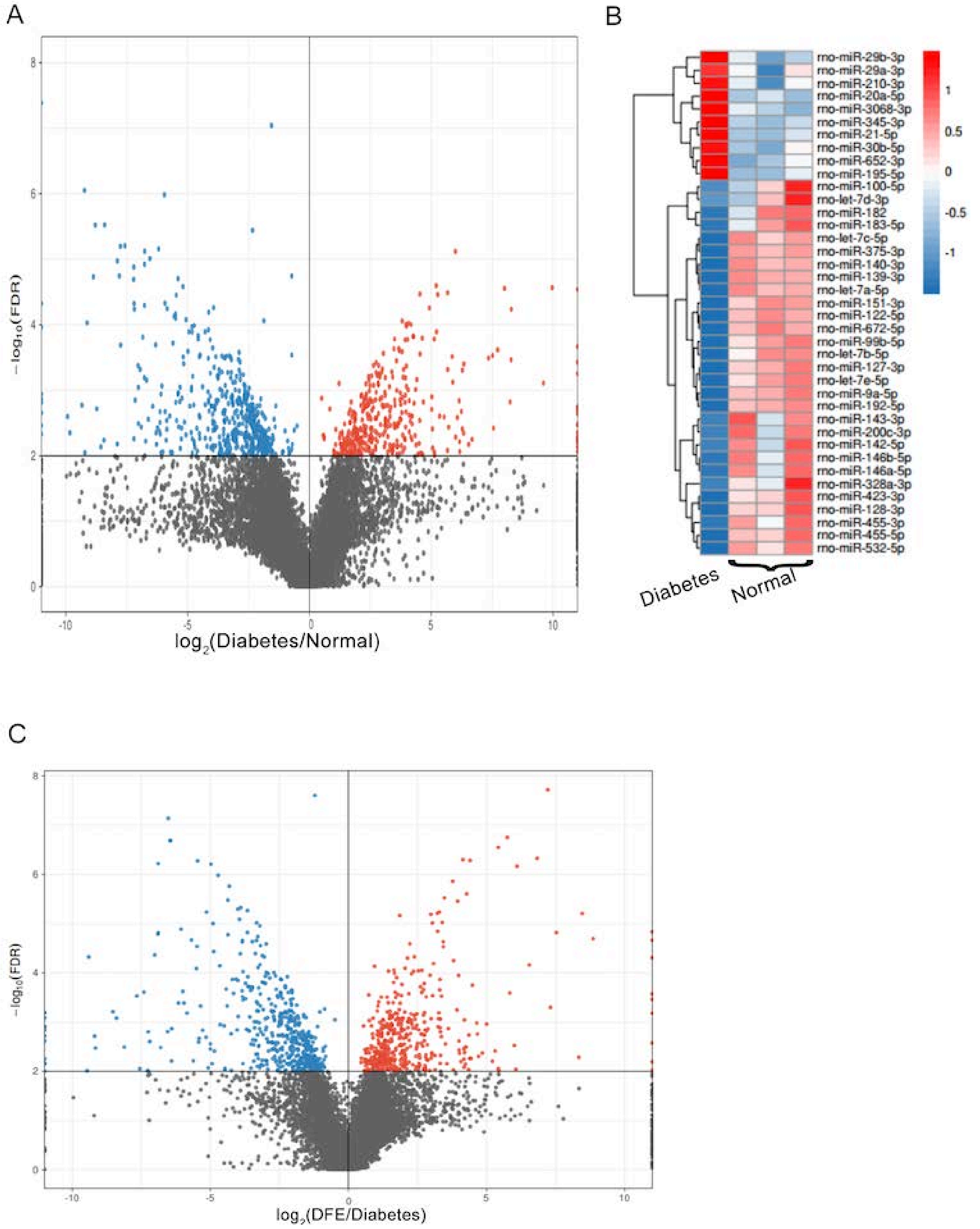

Supplementary Figure 6 | Expression profiling of DEGs and DEMs of the comparisons of diabetes-VS-normal and diabetes-DFE-VS-diabetes in the liver. (A) Volcano plot for the genes in the comparison of diabetes-VS-normal groups; (B) Heatmap of the DEMs in the comparison of diabetes-VS-normal; (C) Volcano plot for the genes in the comparison of diabetes-DFE-VS-diabetes groups.

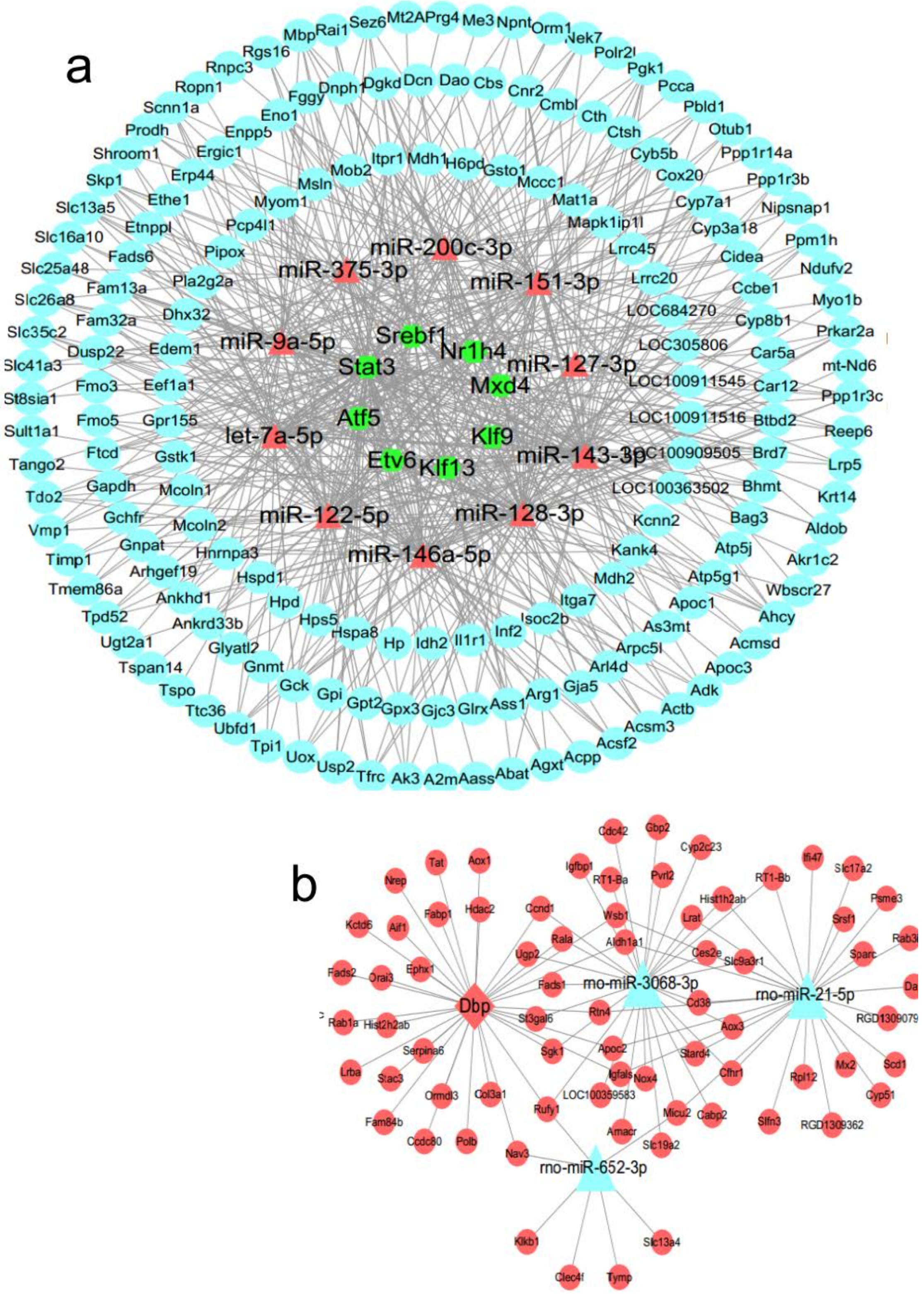

Supplementary Figure 7 | miRNA-TF regulatory network involved in the effects of DFE on alleviating lipid accumulation and hepatic apoptosis in the diabetic liver. Rectangles: TFs; triangles: miRNAs; cycles: NON-TF genes; red color: up-regulated; blue and green: down-regulated.

Supplementary Table 1. Basic statistics of RNA-Seq data for samples.

Sample Name	Tissues/ Treatment	Clean bases (G)	Q20 (%)	GC (%)	Align (%)
A7-1-A	Liver/normal_3	11.32	97.13	49.64	93.64
A9-1-A	Liver/normal_1	11.23	97.06	49.30	93.18
A10-1-A	Liver/normal_2	11.12	97.77	48.99	94.46
B11A	Liver/diabetes_1	10.81	96.42	50.03	94.14
G41A	Liver/diabetes-DFE_1	11.00	97.66	49.59	95.15
G71A	Liver/diabetes-DFE_2	10.82	97.59	49.58	95.34
A1-NA	Pancreas/normal_1	10.21	96.79	54.63	96.43
A2-LA	Pancreas/normal_2	10.04	96.48	53.80	96.06
A6-NA	Pancreas/normal_3	10.10	96.48	54.61	96.51
B32A	Pancreas/diabetes_1	10.81	97.22	49.47	94.52
B62A	Pancreas/diabetes_2	10.80	96.87	48.63	94.30
G42A	Pancreas/diabetes-DFE_1	11.05	97.25	49.34	94.94

Supplementary Table 2. Basic statistics of miRNA-Seq data for samples.

Sample Name	Tissues/ Treatment	Clean Reads (M)	Clean bases (G)	Q20 (%)	GC (%)	Align (%)
A10-1-A	Liver/normal_3	10.59	0.24	99.59	43.93	94.92
A7-1-A	Liver/normal_1	10.50	0.23	99.77	44.32	95.34
A9-1-A	Liver/normal_2	10.53	0.23	99.50	44.03	95.74
B11A	Liver/diabetes_1	10.41	0.23	99.52	46.36	94.21
G41A	Liver/diabetes-DFE_1	10.40	0.24	99.66	46.25	94.32
G71A	Liver/diabetes-DFE_2	10.36	0.23	99.55	44.80	94.08
A1-NA	Pancreas/normal_1	10.54	0.25	99.74	53.99	96.83
A2-LA	Pancreas/normal_2	8.14	0.18	99.85	53.32	96.92
A6-NA	Pancreas/normal_3	10.37	0.24	99.83	54.78	97.34
B32A	Pancreas/diabetes_1	11.98	0.27	99.56	44.53	93.78
B62A	Pancreas/diabetes_2	10.48	0.23	99.58	43.66	96.82
G42A	Pancreas/diabetes-DFE_1	11.95	0.26	99.65	43.77	96.46
